# LAMP-Based Point-of-Care Biosensors for Rapid Pathogen Detection

**DOI:** 10.3390/bios12121068

**Published:** 2022-11-23

**Authors:** Dhrubajyoti Das, Cheng-Wen Lin, Han-Sheng Chuang

**Affiliations:** 1 Department of Biomedical Engineering, National Cheng Kung University, Tainan 701, Taiwan; 2 Department of Medical Laboratory Science and Biotechnology, China Medical University, Taichung 404, Taiwan; 3 Department of Medical Laboratory Science and Biotechnology, Asia University, Wufeng, Taichung 413, Taiwan; 4 Medical Device Innovation Center, National Cheng Kung University, Tainan 701, Taiwan

**Keywords:** loop-mediated isothermal amplification, point-of-care, LAMP-on-a-chip, pathogen detection, biosensors, microfluidic, digital LAMP

## Abstract

Seeking optimized infectious pathogen detection tools is of primary importance to lessen the spread of infections, allowing prompt medical attention for the infected. Among nucleic-acid-based sensing techniques, loop-mediated isothermal amplification is a promising method, as it provides rapid, sensitive, and specific detection of microbial and viral pathogens and has enormous potential to transform current point-of-care molecular diagnostics. In this review, the advances in LAMP-based point-of-care diagnostics assays developed during the past few years for rapid and sensitive detection of infectious pathogens are outlined. The numerous detection methods of LAMP-based biosensors are discussed in an end-point and real-time manner with ideal examples. We also summarize the trends in LAMP-on-a-chip modalities, such as classical microfluidic, paper-based, and digital LAMP, with their merits and limitations. Finally, we provide our opinion on the future improvement of on-chip LAMP methods. This review serves as an overview of recent breakthroughs in the LAMP approach and their potential for use in the diagnosis of existing and emerging diseases.

## 1. Introduction

Point-of-care (POC) testing is performed at the time and location of the healthcare-receiving patients, enabling early-stage diagnosis and prompt medical decisions. Such tests are ideal for environments with limited resources, since they are affordable, need little in the way of equipment, and do away with the requirement for patient follow-up examinations. Nucleic-acid amplification tests (NAATs) play an important role in POC diagnosis by delivering pathogen detections at relatively low concentrations, which allows control of infections spreading at the very early stage [1]. In NAATs, the polymerase chain (PCR) reaction is the most commonly used technique and remains a gold standard. However, the dependence on complex thermal cycling, sophisticated instruments, skilled technical personnel, and long reaction time impedes its use in resource-poor settings and for rapid POC testing. Additionally, the advancement of PCR-based approaches for onsite diagnostics is further hampered by non-specific amplification and a higher risk of false positives [1,2]. Numerous state-of-the-art technologies have been developed in recent years for rapid pathogen detection, such as the enzyme-linked immunosorbent assay (ELISA) [3], surface-enhanced Raman spectroscopy (SERS) [4], surface plasmon resonance (SPR) [5], electrochemical biosensors [6], and clustered regularly interspaced short palindromic repeats (CRISPR) [7]. Although these technologies are promising, they are currently unable to fully address the difficulties associated with pathogen detection, since their implementation in areas with low resources is hampered by the needs for extensive pre-processing, sophisticated equipment, and pricey labelling. There is still a demand for developing integrated biosensors that offer rapid and precise detection of infectious pathogens with high sensitivity [8].

Isothermal nucleic acid amplification tests (iNAATs) have been developed as an alternative and alluring method for highly accurate, quick, and economically efficient nucleic acid amplification [9]. Isothermal amplification (IA) works with a constant temperature (35–65 °C) with a reaction time of 60–90 min. Therefore, it only requires simple hardware for heating and is easy to integrate with a microfluidic device. In addition, IA has higher tolerance toward enzyme inhibitors, making this a robust amplification method [10]. This method includes loop-mediated isothermal amplification (LAMP) [11], recombinase polymerase amplification (RPA) [12], rolling circle amplification (RCA) [13], nucleic-acid-sequence-based amplification (NASBA) [14], and helicase-dependent amplification (HDA) [15]. Among them, LAMP is considered the most promising and robust due to the formation of large copy numbers in a short period (20–30 min) from low concentrations of target molecules. In particular, the amount of amplicon produced from a LAMP reaction is >50-fold more than any other PCR-based amplification technique. LAMP also has the potential to amplify multiple sizes of target DNA ranging from 130–300 bp, delivering amplification for versatile DNA of pathogens [11]. The reverse transcription LAMP (RT-LAMP) technique is employed to amplify RNA molecules using a reverse transcriptase enzyme to synthesize cDNA out of RNA. LAMP has additional advantages compared to other IA; for example: (i) RPA requires a recombinase enzyme, a protein for single-stranded DNA binding, and a polymerase enzyme for strand displacement, but in LAMP, a single DNA polymerase enzyme is used [16]; (ii) LAMP is a one-step reaction process, whereas in RCA, an additional ligation step is required before the amplification [17]; (iii) LAMP amplifies both DNA and RNA molecules—however, NASBA is used to amplify only RNA molecules [10]; (iv) unlike other IA methods, LAMP uses 4–6 primers makes it highly specific [18]; and (v) LAMP also exhibits very high tolerance to polymerase inhibitors, making it highly desirable for direct analysis of body fluids and other clinical samples, without further sample enrichments [19,20]. In addition, the handy design guidelines of LAMP attracted more users, which helped with creating a strong research database over the past two decades. Furthermore, the patent-free status makes LAMP available for the development of commercial products for rapid and onsite diagnosis of infectious diseases at POC.

In the past decade, significant progress has been made in LAMP-based POC biosensors, whereas numerous detection techniques, such as colorimetric, electrochemical, lateral flow, optical, and smartphone-based methods, have been developed for the detection of LAMP amplicons. LAMP-based methods and their applications in food safety have been reviewed previously [21,22], but not in clinical settings specifically. Within the article’s scope, we thoroughly discuss the recent developments of LAMP-based POC modalities for sensing infectious pathogens, plus the detection method of LAMP amplicons based on the end-point and real-time monitoring. The advantages and disadvantages of LAMP-based systems and the lab-on-chip (LOC) design are also demonstrated and clarified by examples for each method.

## 2. Overview of the LAMP Assay

### 2.1. Principle

**Figure 1:**
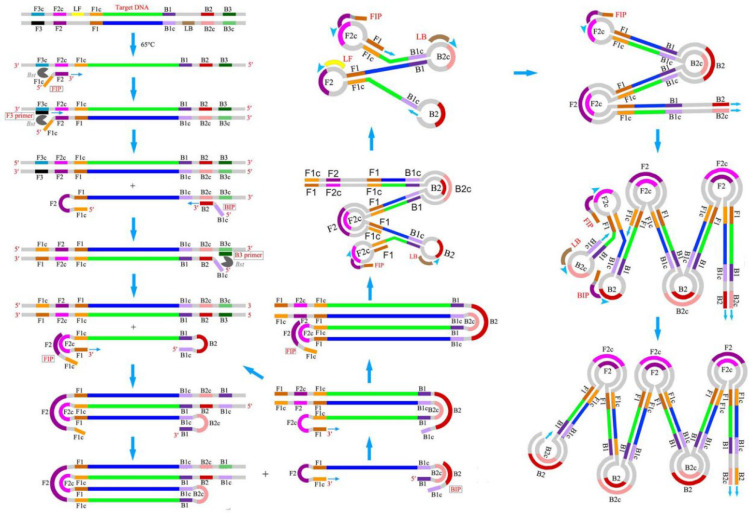
Schematic of LAMP mechanism. Reproduced with permission from reference [24], open access. Copyright 2016 Frontiers Media.

Isothermal amplification (IA) is a simple, rapid, and efficient method that can accumulate nucleic acid at a constant temperature without using additional advanced high-tech equipment. Due to the operational simplicity, IA methods are easy to integrate with microfluidic chips to develop rapid, sensitive, and onsite POC diagnostic tools [9]. Among the IA methods developed during 1991–2006, LAMP has risen as a rapid, robust, and highly specific nucleic acid amplification technique with the ability to generate a large number of products within a short time in a single-step reaction. LAMP was first reported in the year 2000 by Notomi et al. [11], and since, it has gained popularity in research, clinical, and industrial applications and emerged as an alternative to conventional PCR by eliminating the dependence on an expensive thermocycler. Additionally, the high tolerance of LAMP towards the enzyme inhibitors makes it a suitable candidate for onsite detection of clinical and biological samples without any sample enrichment. Unlike conventional PCR, LAMP requires four specific primers targeting six distinct regions (F3c, F1c, F2c, B2c, B1c, and B3c) on the DNA, providing enhanced specificity. This set of primers includes two inner primers called forward inner primer (FIP) and backward inner primer (BIP), whereas F3 and B3 are two outer primers, also known as “displacement primers” [23]. Figure 1 displays the mechanism of the LAMP reaction [24]. Both the inner primers FIP and BIP consist of hybridization sequences, F2 and F1c and B2 and B1c, respectively. The inner primers contain a unique “fold back” arrangement that creates stem–loop motifs for self-priming [11]. LAMP amplification can be split into two steps, i.e., the structure creation and the cyclic amplification. LAMP benefits from a DNA polymerase with strand-displacing activity, which avoids the need for a complex heat cycling step for dsDNA denaturation between each amplification cycle. In the first step, the F2 of the inner primer binds with the F2c of the target stand, initiating DNA synthesis due to the polymerase activity. Next, the F3 of the outer primer binds with F3c, followed by the displacement of the freshly synthesized DNA and the release of the target DNA. The released strand containing complementary F1 and F1c regions gives rise to a stem–loop structure. In the 3′ ends, another stem–loop structure will be formed under a similar mechanism to BIP and B3, forming a “dumbbell”-like structure. This “dumbbell structure” will undergo self-priming and serve as a starting material for the cyclic amplification step. As a result, LAMP produces a significant number of amplicons in a rapid period. Due to these characteristics, LAMP is well-suited for usage in POC biosensors that feature simplicity, resilience, miniature, and ease of operation.

### 2.2. Primer Design

In addition to the four basic primers (FIP, BIP, F3, and B3), LAMP utilizes two other loop primers, the forward loop primer (LF) and backward loop primer (LB) [18], for the acceleration of the amplification and reduction of the time to one-third of the usual. The loop region is typically situated between B1–B2 and F1–F2 of the dumbbell structure, providing additional initiation sites for LAMP. The basic principles of LAMP primer designs are as follows: (i) there should be a minimum distance of 40–60 bases between F2 and F1c and B2 and B1c for enough space to generate loop primers; (ii) repeats of dinucleotides (e.g., AGAGAG) and repetition of a single nucleotide more than three times (e.g., ATTTT) should be avoided, as it can cause a mispriming; (iii) three or more runs of Gs should be avoided, as it may raise an issue with primer synthesis and purification; (iv) to avoid the secondary structure formation, the 3′ ends of the designed primers should not be AT-rich or complementary to one another; (v) the Tm for the primer pairs (e.g., F2 and B2) should be similar to a maximum temperature difference of 5 °C, and the F1c and B1c should have a higher Tm than F2 and B2 for the immediate formation of the stem–loop structures; (vi) in the case of GC-rich primers, the melting temperature (Tm) should be about 60–65 °C, and in the case of AT-rich primers, it should be around 55–60 °C. A relatively higher Tm is required for GC-rich primers due to the formation of a higher number of hydrogen bonds. The Tm of the primers is calculated using Equation (1); (vii) to obtain higher stability of the primers, the ΔG values of the 5′ ends of F1c/B1c and 3′ ends of F2/B2 and F3/B3, should be less than −4 kcal/mol; and (viii) to eliminate the formation of secondary structures, the 3′ ends should not be AT-rich [25]. NUPACK software (version 4.0, created by Prof. Niles A. Pierce and his team, Copyright© 2006–2022 California Institute of Technology, http://www.nupack.org/partition/new, accessed on 20 July 2022) is used to analyse putative secondary structures, and primer dimer production and hybridization stability [26]. In addition to that, reports suggest that the integration of poly T linkers (TTTT) between F2 and F1c of FIP and between B2 and B1c of BIP can improve the reaction speed by loop formation [27].

(1) $$T_{m}=\frac{\Delta H}{\Delta S{}^{\circ}+R\cdot ln\left(\frac{C_{T}}{X}\right)}-273.15$$

where *R* is the gas constant 1.9872 × 10^−3^ kcal K^−1^ mol^−1^; the number in the second term, −273.15, is a conversion factor from K to °C; *C_T_* is the total molar concentration (M) of the strands; and *X* is considered four for non-self-complementary duplexes.

Primer explorer V5 (http://primerexplorer.jp/elamp4.0.0/index.html, accessed on 20 July 2022) is the most commonly used software for LAMP primer design. The selected target region should be 200–2000 bp in length, and the saved sequence can only be uploaded in FASTA, .txt, or GenBank format. The steps for designing LAMP primers using Primer Explorer V5 are as follows: (i) launch the program, upload the correct format of the sequence file, and click on “Primer Design” to display the regions of the target sequences; (ii) click on “Generate” to get the designed primers of the best five primer options for a particular gene sequence; (iii) choose the primer set with the highest ΔG value for dimerization and click on “Display” to exhibit the designed primer list in a new window; (iv) select the primer with a ΔG value of ≤−4 kcal/mol at the 5′ end of F1c/B1c and at the 3′ end of F2/B2 and F3/B3 and click on “Confirm” to obtain the particular primer details; (v) after choosing the appropriate primer set, click on “Primer information” to save the file for loop-primer generation, and then click on “Save” to download the primer sequences in a Word/Excel document; (vi) to generate the loop primers, re-launch the software and upload the saved file from step (v); (vii) click on “Primer Design” to display the LF and LB in the target sequence; (viii) next, click on “Generate,” followed by “Display,” to obtain the designed LF and LB primers in a new window; (ix) choose the loop primer pair with the highest ΔG value for dimerization and click on “Confirm” to display the primer details; (x) finally, choose the loop primers with lowest ΔG value at 3′ end and click on “Save” to download the primers. BLAST tool (www.ncbi.nlm.nih.gov, accessed on 20 July 2022) is used to check the specificity of each of the designed primers. The designed primer should display high specificity for the targeted gene sequence. The software also provides advanced settings with the flexibility of choosing AT and GC-rich options and the desired Tm to design target-specific customized primers. Nevertheless, it is not advised to change the lengths of primers. Primer explorer V5 is an easy-to-use online tool for LAMP primer design; however, it has some limitations—e.g., (i) simultaneous design of the loop primer is not possible; (ii) it only allows a gene sequence up to 2000 bp; (iii) besides ATGC, it does not support any other IUPAX characters; and (iv) it allows only one execution process.

Apart from the primer explorer, multiple online primer design software tools are available, such as Premier Biosoft, Optigene LAMP designer, NEB LAMP primer designer tool, and more. Several methods have been published in recent years for designing target-based primers. For example, Zhang et al. [28] developed a novel Python script for designing LAMP-based variant-specific probes to detect cancer mutations, evaluated using the sequences of *ESR1 p.E380Q* and *ESR1 p.Y537S* cancer-specific mutations, as the algorithm generates the two best primer sets for the respective target with an input of the DNA sequence. In another work, Jia et al. [29] presented a whole genome-based LAMP primer design (GLAPD) tool, which can be downloaded using the following website: http://cgm.sjtu.edu.cn/GLAPD/ or https://github.com/jiqingxiaoxi/GLAPD.git (accessed on 20 July 2022) and http://cgm.sjtu.edu.cn/GLAPD/online/ (accessed on 20 July 2022). It is a novel approach that uses complete genomes to create LAMP primers for a set of target genomes for rapid detection of foodborne pathogens, which yielded similar results to Primer explorer V5 with the same nucleotide sequences. Additionally, a theoretical approach was developed by Savonnet et al. [30], who studied the dynamics of LAMP’s second stage from the fundamentals of reaction rates to deduce physio-chemical properties from the experimental signal development. The dumbbell structure used in the study skipped the first stage of the LAMP. This model anticipates that the concentration of amplification products will rise according to a logistic function.

### 2.3. Features for Rapid Pathogen Detection

Due to the formation of high copy numbers (~10^9^) in a short period (<1 h), LAMP has been considered one of the most efficient and popular diagnostics tools used for rapid pathogen detection. The inner primer plays a crucial role in LAMP, as it has brought a lot of benefits for making LAMP a robust, rapid, and advanced amplification method. Essentially, the strand displacement of the inner primer occurs at a constant temperature (60–65 °C), eliminating the need for complex thermal cycling and making LAMP a suitable candidate for POC devices with its simple design and rapidness. The stem–loop structure provides amplification based on self-priming, allowing the production of large numbers of amplicons independent of template DNA strands. Hence, this produces a high copy number of DNA in a short period, enabling rapid target detection with very high sensitivity. LAMP provides a high tolerance towards the inhibitors, reducing the effort of sample pre-treatment. Therefore, the ideal of a simple LAMP-based “sample-in-result-out” POC device is feasible. Compared to other methods, LAMP uses 4–6 different primers targeting six distinctive regions, making it highly specific. A mismatch with any target sequences will lead to a negative amplification, eliminating the chances of non-specific amplification. It is reported that the LAMP assay may possess higher sensitivity compared to the gold-standard PCR. It can have a detection limit as low as five copies per reaction [31]. Hence, LAMP can be used for early disease diagnosis and to contain the spread of infectious pathogens. Furthermore, the production of pyrophosphate molecules during LAMP enables visual identification of the target, making the detection method easy and simple without using any expensive instrumentation. As another factor, LAMP produces polymerized target DNA with various base pair lengths from 300 to 25 kb [11]. This large number of amplified nucleic acids could increase the viscosity of solutions. This micro-level viscosity change can be detected by an advanced and ultrasensitive particle-imaging technique called rotational diffusometry, bringing down the detection time to less than 10 min [32]. A detailed discussion is included in Section 3.1.5.

Another fascinating tool named CRISPR/Cas system [33] has been integrated with LAMP for rapid detection of pathogenic nucleic acids (NA) with very high sensitivity [34]. First, the Cas protein (Cas9, Cas12, and Cas13) forms a complex with a special RNA sequence called CRISPR-RNA (crRNA), activated once it binds to the specific target RNA/DNA, triggering the cleaving mechanism of Cas protein. Subsequently, this activated complex cleaves the reporter probe carrying a fluorophore and a quencher, releasing significant fluorescence. The detection can be performed via monitoring fluorescence and in lateral-flow strips [35,36]. Recently, CRISPR, in combination with RT-LAMP, has been investigated for rapid and highly accurate POC diagnosis of COVID-19 [37,38]. Due to the simplicity of LAMP operation, these ultra-sensitive and highly accurate state-of-the-art detection techniques are integrated easily, making LAMP a rapid and sensitive pathogen detection tool. In the next section, we discuss the various end-points and the real-time detection methods for monitoring LAMP reactions.

## 3. LAMP Detection Methods

### 3.1. End-Point Detection

#### 3.1.1. Colorimetric Detection

Colorimetric detection of LAMP products can be divided into two categories: turbidity and use of dye. During the LAMP reaction, deoxyribonucleotide triphosphate (dNTPs) reacts with magnesium sulphate (MgSO_4_) to yield a white precipitate of magnesium pyrophosphate (Mg_2_P_2_O_7_), increasing the solution’s turbidity [39]. No additional read-out instrument is required, as the change in turbidity can be determined by the human eye. Garg et al. [40] introduced successful LAMP-based turbidimetric detection of *Mycobacterium leprae* targeting the 16s rRNA gene. Likewise, similar studies reported the detection of multiple pathogens—for instance, *Clostridium botulinum* [41], *Streptococcus pneumoniae* [42], and *Streptococcus agalactiae* [43]. However, this method provides low sensitivity, as the faint product can easily be misjudged.

**Figure 2:**
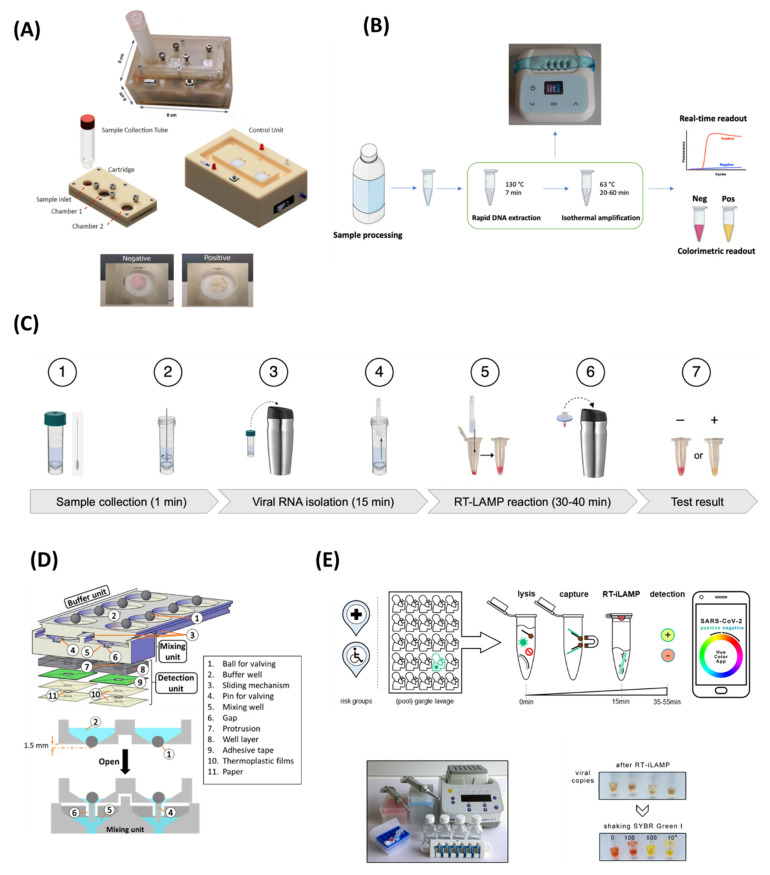
LAMP-based biosensors using dye detection. (**A**) A self-contained device diagnosis of SARS-CoV-2 virus with a disposable cartridge and an Arduino-based reusable microcontrolling unit. (**B**) Schematic representation of *Clostridium tyrobutyricum* in milk at POC. (**C**) Illustration of a thermos-based home test for COVID-19. (**D**) Polycarbonate POC testing device with a ball-based valve system for sample delivery and reagent preparation. (**E**) Workflow of Cap-iLAMP. Reproduced with permission from references [49,51,53,60,61]. Copyright 2021 and 2022 Springer Nature, 2021 American Chemical Society, 2021 MDPI.

As alternatives to the turbidity method, molecular probes are employed for the colorimetric detection of LAMP products. The most common colorimetric indicators are metal-ion indicator dyes, namely, calcein, hyroxynapthol blue (HNB) plus eriochrome black T (EBT), pH-sensitive dyes (phenol red, neutral red), and other fluorescence probes. These dyes provide a significant change in colour with the presence of LAMP amplicons but do not inhibit the amplification process [44]. Therefore, they can be added at the beginning of the LAMP reaction. Additionally, various DNA intercalating dyes, such as SYBR Green I, propidium iodide (PI), and SYTO-81, are used to detect the LAMP product with high sensitivity and can be easily monitored under visible light or UV regions. HNB and calcein are the most commonly used dsye for the colorimetric detection of LAMP. Initially, calcein binds with Mn^2+^ ions, which quenches its fluorescence activity, and the LAMP reaction mixture appears orange in colour. Then, Mn^2+^ ions form a complex with pyrophosphate P_2_O_7_^4−^ during the amplification process, hence recovering the green fluorescence. In addition, the fluorescence signal is further enhanced by calcein–Mg^2+^ complex formation [39]. Similarly, HNB shows a change in colour from purple to blue due to the reduction in Mg^2+^ ions’ concentration [44]. Zhu et al. [45] developed a capillary-array-based POC platform combining LAMP for the detection of the African swine fever virus (ASFV) by targeting five different genes (D1133L, B962L, B646L, G1340L, and C717R). The capillary array is named “Hive chip” due to its hive-like shape, where the reagents are loaded and distributed inside the chip by capillary forces. Direct blood samples treated with sodium hydroxide were used for LAMP without any further DNA extraction and showed a LOD of 50 copies/µL over an assay time of 70 min by using calcein dye. In another work, Yao et al. [46] designed a PDMS-made, portable, self-driven microfluidic chip enabling automatic and rapid loading of samples for RT-LAMP. The system can detect eight vector-borne viruses, including Chikungunya virus (CHIKV) and Dengue virus (DENV). Calcein-based colorimetric detection achieved a LOD of 100 copies for CHIKV and DENV-1 in just 50 min, including 3 min for self-powered sample loading and 45 min for RT-LAMP. Furthermore, the developed method tested using 120 mosquito samples and 50 clinical samples provided consistent results with RT-PCR analysis. Similarly, another self-driven POC microfluidic platform was developed as a combination with LAMP for rapid colorimetric detection of blood-borne pathogens empowered by calcein. Reagents can be loaded into the self-driven chip within 12 min. After 50 min LAMP, results were recorded and monitored using a smartphone camera. Multiplex detection of three pathogens, namely, human immunodeficiency virus (HIV), hepatitis B (HBV), and hepatitis C virus (HCV), with a LOD of 2 copies/µL, was achieved with a turnaround time of 65 min [47]. Jin et al. [48] devised a self-priming compartmentalization (SPC) microfluidic platform for HNB-based naked-eye detection of food-borne pathogens. The self-filling of the microfluidic LAMP platform was achieved by negative pressure. On top of that, the closed wells eliminated cross-contamination to ensure accuracy. Simultaneous detection of six food-borne pathogens was realized through the SPC device with a detection limit of 10^2^–10^3^ CFU/mL in an assay time of 60 min. In another effort, Deng et al. [49] reported a LAMP-based, portable, and self-contained device for POC diagnosis of the SARS-CoV-2 virus. A disposable cartridge consisting of a PDMS-based microfluidic chip was designed to perform reagent transportation, viral lysis, and a RT-LAMP reaction (Figure 2A). Microchip operation was controlled by a battery-operated, Arduino-based microcontroller with a size of 6 × 9 × 4 cm^3^. The pocket-size device obtained colorimetric detection of 300 copies of RNA per reaction with a sample-to-answer time of 30 min.

During LAMP, a pH-sensitive dye can sense the amplified DNA molecule through the solution’s pH, which becomes lower due to the products [50]. For example, a POC diagnostic method was developed for rapid and sensitive detection of *Clostridium tyrobutyricum* in milk using pH-sensitive Cresol Red dye (Figure 2B). DNA extraction was achieved by chemical lysis, which eliminates the time-consuming multistep purification process. A LOD of ~2 spores/mL was achieved by direct visualization of colour change from red to yellow in contaminated milk samples with high sensitivity and specificity [51]. Ye et al. [52] developed a glass fibre paper-based POC diagnostic tool capable of RNA extraction in 5 min, the isothermal amplification of the target gene in just 25 min, and a visual readout of the final product. This low-cost paper disc obtained a LOD of 1 × 10^3^ copies/mL for rotavirus A with very high specificity by employing pH-sensitive neutral red dye. Furthermore, this paper device was tested with 48 clinical samples and showed a comparable detection sensitivity to the gold-standard, RT-PCR. In another study, Song et al. [53] developed rapid, low-cost, easy-to-use self-testing kits for COVID-19 by direct naked-eye detection of colorimetric test results (Figure 2C). The complete workflow, including sample collection, RNA purification, isothermal amplification of target genes in a thermos, and visual analysis of results takes less than 60 min. The following lyophilization protocol allows long-term storage of RT-LAMP reagents at room temperature for 10 days. A LOD of 100 RNA copies/reaction was achieved with 99% specificity.

The dye SYBR Green I, which exhibits a visible colour change under white light (reddish-orange to yellowish-green) and fluoresces in the ultraviolet regions has been used in the past for monitoring LAMP reactions. For example, Wang et al. [54] reported the detection of *Fusarium proliferatum* with a LOD of 10 pg/µL in 30 min. Additionally, a SYBR Green I-based LAMP assay was used for the detection of *Listeria monocytogenes* (*L. monocytogenes*), [55] *Enterocytozoon hepatopenaei*, [56] Milk vetch dwarf virus [57], and *Bacillus anthracis* [58]. Recently, Oliveira et al. [59] reported RT-LAMP-based detection of SARS-CoV-2 in a fully integrated and automated POC device. SYBR green I was used for the colorimetric detection of the viral genome with a LOD of 10^−3^ copies/reaction. Clinical samples tested using this developed protocol correlated well with real-time qPCR, demonstrating a reliable tool for rapid COVID-19 diagnosis in resource-limited regions. In another effort, an RT-LAMP integrated polycarbonate-based POC testing device was developed for multiplex detection of SARS-CoV-2 and influenza A H1N1 virus within 50 min (Figure 2D). This multiplexed platform allows reagent mixing and preparation without any power outlets or pipetting. A ball-based valve system was used for sample delivery, enabling paper-based RNA enrichment and reagent preparation. Colorimetric detection for SARS-CoV-2 and H1N1 virus was achieved with LODs of 2 and 6 genomes per reaction, respectively. Furthermore, this device can detect virus particles from clinical and environmental samples [60]. Bokelmann et al. [61] presented the Cap-iLAMP acronym for capturing and an improved loop-mediated isothermal amplification method for smartphone-based colorimetric detection of SARS-CoV-2 from gargle lavage samples (Figure 2E). This bulk testing method utilizing the hybridization capture for RNA purification enables a detection sensitivity of 500 copies/reaction within 55 min. Cap-iLAMP eliminates false positives by allowing single-positive specimens to be identified in pools of 25 negative samples, lowering the reagent cost for each test to less than ~1 Euro per person. Colorimetric detection eliminates the use of expensive readers and signal processing units, making it low-cost and easy to operate. However, it is qualitative, and obtaining quantitative analysis remains challenging.

#### 3.1.2. Electrochemical Detection

Electrochemical sensors have demonstrated promising benefits, such as rapid detection with high sensitivity and selectivity for developing POC diagnostic tools [62,63]. They have been extensively explored for the detection of single nucleotide polymorphisms (SNPs) [64,65], nucleic acids, and their amplified products over the past few decades [66,67,68]. Integration of LAMP with electrochemical sensors can provide fast and ultra-sensitive detection of various analytes by measuring the oxidation reduction in the electro-active molecules due to the interaction with the LAMP product. Another strategy involves the immobilization of the probe on the surface of the working electrode with a redox reporter, which can monitor the electrochemical signals by linear-sweep voltammetry (LSV) [69], square-wave voltammetry (SWV) [70], differential-pulse voltammetry (DPV) [71], or conductivity [72]. The LAMP product can be detected after the reaction or monitored in real-time through electrochemical sensors [73]. Regarding real-time detection, numerous redox molecules, including methylene blue (MB) and ruthenium hexamine, can be used, as they do not inhibit DNA amplification [21]. However, some of them, namely, Hoechst 23458, may impede the LAMP reaction and are unusable for instantaneous sensing [74].

**Figure 3:**
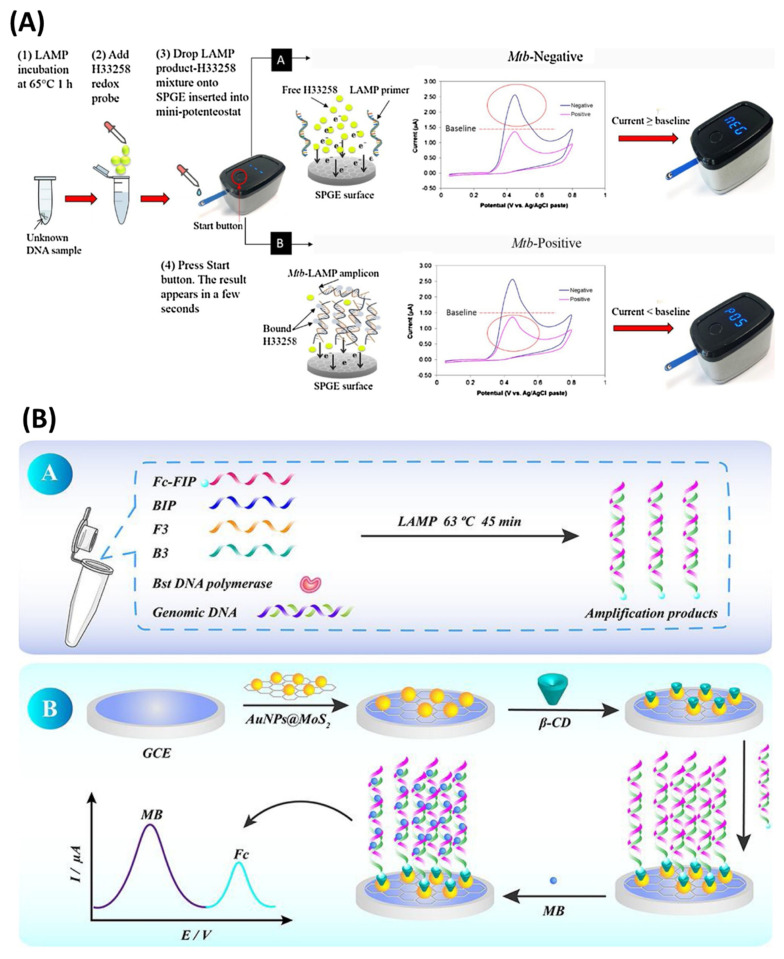

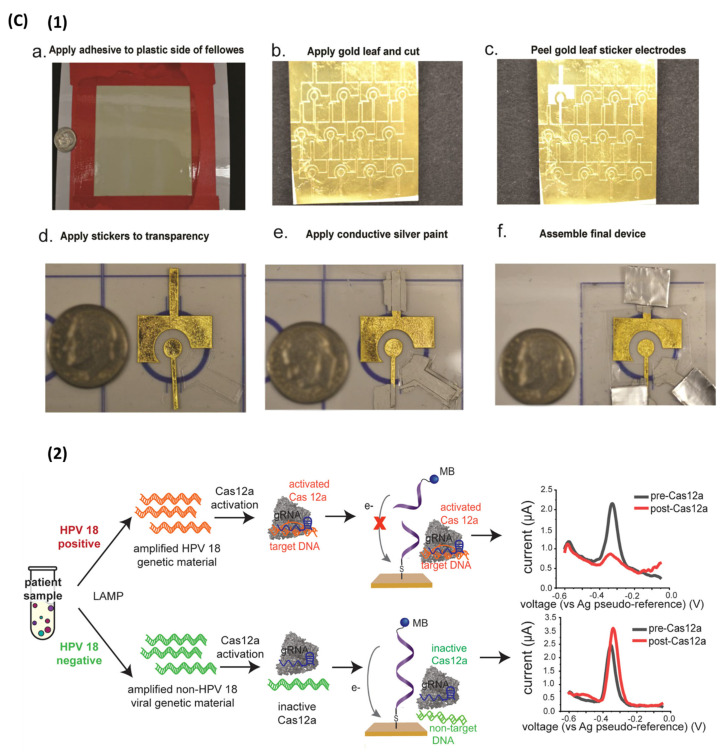
LAMP−based electrochemical sensors. (**A**) LAMP amplicon detection on a screen-printed graphene electrode (SPGE) using a portable potentiostat device. (**B**) Schematic illustration of LAMP E-sensor for detection of Group B *Streptococci*. (**C**) (**C1**) Gold-leaf electrode’s fabrication; (**C2**) Working principle of CRISPR/Cas12a activation-based sensor for detection of the HPV virus. Reproduced with permission from references [75,77,78]. Copyright 2020 Elsevier, 2021 American Chemical Society.

Jaroenram et al. [75] detected the LAMP amplicons on a screen-printed graphene electrode (SPGE) using an in-house mini potentiostat device to measure the cyclic voltammogram displayed on an LCD screen with Hoechst-33258 as the electrochemical redox agent (Figure 3A). The assay had a LOD of 40 CFU/reaction for *Mycobacterium tuberculosis* with a turnaround time of 65 min. A similar electrode system and a portable potentiostat device were used to detect the LAMP product of *Vibrio parahaemolyticus* (*V. parahaemolyticus*) with a detection limit of 0.3 CFU per 25 g of raw seafood in 45 min [76]. In another effort, Fu et al. [77] developed a LAMP-based electrochemical sensor for Group B *Streptococci* (GBS), which demonstrated a broad detection range of 1 fg µL^−1^ to 100 pg µL^−1^. Thiolated β-cyclodextrin (SH-β-CD) was immobilized onto the AuNPs@MoS_2_-modified glassy electrode surface via Au–S bonding. Thus, the ferrocene (Fc)-modified primers enable the binding of the amplified product via a host–guest interaction between Fc and β-CD (Figure 3B). MB was used as a redox agent to produce a strong electrochemical signal during the interaction with the LAMP product, yielding a low detection limit of 0.23 fg µL^−1^. In another study, Zamani et al. [78] introduced a CRISPR/Cas12a activation-based sensor for the human papillomavirus (HPV) virus, which achieved a LOD of 10^4^ copies of DNA per reaction. A methylene-blue-tagged oligo was immobilized on a gold-leaf electrode (Figure 3C). Square-wave voltammetry (SWV) was used for electrochemical readouts of methylene-blue-tagged oligos by Cas12a enzyme activity. Other studies employed a combination of digoxigenin-labelled nucleotides (DIG-dUTPs)-based LAMP and streptavidin-modified magnetic beads for the separation and amperometric detection of HPV16 and HPV18 in a carbon-based electrode system. After a successful LAMP reaction, the resulted amplicons were incubated with the modified magnetic beads, followed by anti-DIG Ab conjugated with horseradish peroxidase (HRP). The magnetic beads were concentrated on the electrode’s surface using a magnet, and amperometric monitoring of the HRP reaction was performed [79]. In the next attempt, 61 cervical samples for both genotypes were screened and validated with real-time PCR and commercial HPV tests such as INNO-LiPA and COBAS. The system had 90% accuracy [80]. Recently, Ramírez-Chavarría et al. [81] proposed a LAMP-based low-cost electrochemical sensor for specific detection of a SARS-CoV-2 gene sequence, in which RT-LAMP is performed on a disposable test strip consisting of a screen-printed electrode. Methylene blue was used as an electrochemical redox probe to deliver a diffusion-controlled current during the interaction with LAMP products, as square-wave voltammetry (SWV) was chosen for electrochemical readouts. The test strips showed a detection sensitivity of ~2.5 × 10^−6^ ng/µL, along with high specificity and reproducibility.

#### 3.1.3. Lateral Flow Assay Detection

**Figure 4:**
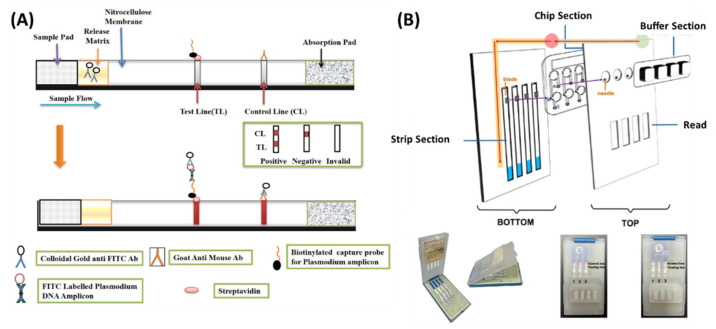
LAMP-based lateral flow biosensors. (**A**) Outline of the lateral flow strip for detection of LAMP amplicons. (**B**) A portable microfluidic platform integrated with a pregnancy test strip (PTSs) for POC diagnosis of COVID-19. Reproduced with permission from references [90,92]. Copyright 2021 American Chemical Society, 2022 MDPI.

Lateral flow tests (LFTs) were used extensively in recent years for LAMP-based rapid POC diagnostics tests. The test line on the lateral flow assay strip catches biotin-labelled LAMP amplicons hybridized with fluorescein isothiocyanate (FITC)-labelled DNA probes, which later bind to gold-based anti-FITC antibodies to create a legible output. Non-hybridized FITC probes attach to gold-labelled anti-FITC antibodies, generating a biotin-free double complex that travels from the test to the control line. The detection sensitivity with extracted genomic DNA was 600 fg/reaction for *Mycoplasma pneumoniae* [82], 100 fg/reaction for methicillin-resistant *Staphylococcus aureus* (MRSA) [83], and *Pseudomonas aeruginosa* [84] 630 fg/reaction for *V. parahaemolyticus* [85] and 13.5 fg/µL for *Salmonella* [86]. For obtaining a higher sensitivity and accuracy, nanomaterials and enzymatic enhancements were investigated by various research groups. For example, Shi et al. [87] developed a propidium monoazide (PMA) based nanozyme strip for viable detection of *L. monocytogenes* with a LOD of 10 CFU/mL. The color change was further enhanced by an enzymatic reaction between the nanozyme and DAB/H_2_O_2_ (enzyme substrate). Gold nanoparticles were replaced by Fe_3_O_4_ nanoparticles, which provided better dispersibility. In another work, A quantum dot nanobead-based LFT was developed by Shang et al. [88] for quantitative detection of *S. typhimurium* (*S. typhimurium*). The visual LOD of 10^3^ CFU/mL achieved a linear range of 10^2^–10^8^ CFU/mL, 10-fold higher than AuNPs labelled strips. Yang et al. [89] reported polymer nanoparticles lateral flow biosensors in combination with LAMP for the detection of *Brucella abortus* targeting *BruAb2_0168* gene. The assay achieved a LOD of 100 fg/reaction with a turnaround time of 85 min. Additionally, a multiplex LAMP-based LFT was reported for the detection of two malaria-causing parasites, *viz.*, *Plasmodium vivax* and *Plasmodium falciparum*, where FITC and digoxigenin-labelled probes hybridized with the LAMP amplicons having a complementary nucleotide sequence (Figure 4A). A clear band was visualized on the strip within 60 min for both species. Detection sensitivity reached 0.15 pg/µL for extracted genomic DNA [90].

LFT combined with RT-LAMP has been proven to be a promising tool for rapid diagnosis of COVID-19 at POC. A polymerase-nanoparticle-based LFT coupled with RT-LAMP was designed by Zhu et al. [91] for the simultaneous detection of specific genes from the SARS-CoV-2 genome using two sets of LAMP primers in a single reaction tube. The primers for ORF1ab and N genes were labelled with FITC and digoxin, respectively, with an additional biotin tag. Immunoreaction between FITC, digoxin, and biotin-labelled duplex LAMP amplicons with anti-FITC/digoxin in the test line produced a characteristic crimson band that enables the multiplex detection of both genes with a LOD of 12 copies/reaction. The entire process, from sample collection to the results, was accomplished within 1 h. In another work, a palm-sized, portable microfluidic platform integrated with a pregnancy test strip (PTSs) was developed for POC diagnosis of the SARS-CoV-2 genome (Figure 4B). Highly specific human chorionic gonadotropin probes (hCG-p) were designed to hybridize with the specific loop region of the RT-LAMP amplicons, and thus to be unable to move through the PTSs and appear only on the control line on the strip. Ultrasensitive detection with a LOD of 0.5 copy/µL was achieved with an assay time of 120 min by on-site naked-eye visualization [92]. LAMP provides specific amplification of the target, and lateral flow sensors offer a cheap and easy readout method. Therefore, LAMP combined with LFTs has enormous potential for creating POC diagnostic platforms. This technique can be combined with microfluidic platforms to allow high-throughput analysis while removing the possibility of cross-contamination.

#### 3.1.4. Optical Detection

**Figure 5:**
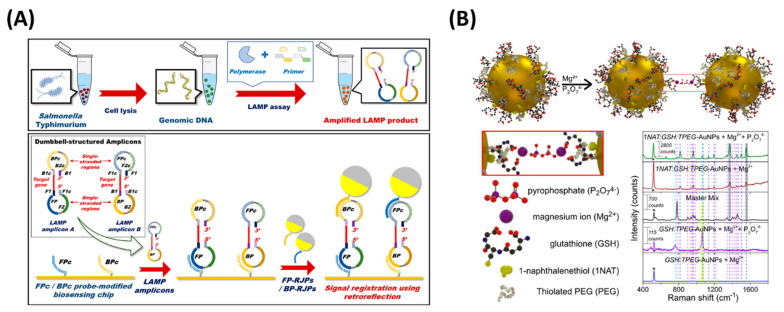
Optical detection methods of LAMP amplicons. (**A**) Schematic representation of retroreflective Janus particle−based biosensor. (**B**) Detection of LAMP amplified DNA using SERS. Reproduced with permission from references [96,101]. Copyright 2018 American Chemical Society, 2020 MDPI.

Optical devices have emerged as an alternative solution and are extensively used in the end-point detection of LAMP products. Optical sensors and instruments, such as a complementary metal-oxide semiconductor (CMOS) [93], surface plasmon resonance (SPR) [94], and surface-enhanced Raman scattering (SERS) [95], have been used for monitoring LAMP amplicons. Chun et al. [96] designed a detection method for food-borne pathogens using a CMOS sensor and white LED light by integrating retroreflective Janus particle (RJP) and LAMP, which achieved a LOD of 10^2^ CFU for *S. typhimurium*. A DNA probe immobilized on the RJPs was injected into the LAMP product, resulting in a hybridized sandwich structure formation between the LAMP product and RJPs (Figure 5A). In another work, a smartphone-based image processing method was applied in sensing food-borne pathogens using SYTO-9, which yielded a LOD of 6 copies/µL for *L. monocytogenes* [97].

Surface plasmon resonance is an optical method used in the past for LAMP detection [94]. LAMP, combined with SPR imaging, was reported by Nawattanapaiboon et al. [98] for the detection of MRSA in clinical samples. A bio-functionalized array was fabricated by immobilizing biotinylated probes using self-assembled monolayer surfaces (SAMs), which allowed multiplex detection of *fem*B and *mec*A genes of MRSA and obtained a LOD of 10 copies per microliter sample. In another effort, gold nanoprobes, complementary to F2 and F1C, were modified for SPR-based detection of LAMP [99]. This assay achieved a detection limit of 20 CFU/mL for *Salmonella typhi* in 30 min.

Over the past few years, LAMP-integrated surface-enhanced Raman scattering (SERS) gained tremendous popularity in nucleic acid amplification tests (NAATs) due to its high sensitivity and reproducibility [95]. Draz and Lu [100] reported a LAMP-combined SERS assay for the rapid and sensitive detection of *Salmonella enterica* (*S. enterica*), in which the Raman active gold nanoprobes consisted of modified AuNPs with cy5/DNA probes for SERS detection of the LAMP products. The assay achieved a detection limit of 66 CFU/mL for *Salmonella enteritidis*, showing 100-fold higher sensitivity than conventional PCR. In addition, the assay expressed high specificity, as the detection of *Salmonella* was validated from contaminated milk samples. In another work, a microdroplet-based LAMP was paired with SERS to detect pathogens from the food matrix [101]. As displayed in Figure 5B, multifunctional AuNPs consisted of the following components: Raman reporter 1-naphthalenethiol, with glutathione (GSH) as a complexing agent and thiolated polyethylene glycol as a stabilizer. Due to the complexation of the carboxylic acid group of glutathione and pyrophosphate, the AuNPs aggregated and were then used to detect the target, which provided an enhanced SERS signal, resulting in the detection of *L. monocytogenes* with a sensitivity of 3.6 × 10^2^ CFU/mL in the milk sample.

#### 3.1.5. Diffusometric Method

The combination of LAMP with diffusometry, a technique based on the simple principle of Brownian motion of microscopic particles, has emerged in recent years as a method of sensitive and rapid detection of infectious pathogens [32]. According to the Stokes–Einstein equation, particle diffusion is inversely proportional to the sample viscosity. Therefore, the microlevel viscosity change due to the presence of LAMP amplicons is determined by analysing the series of particle images. Clayton et al. [102] reported the LAMP combined particle diffusometry method for the detection of blood-borne pathogens, as 200 nm fluorescent polystyrene particles were imaged with a CCD camera and analysed with a cross-correlation algorithm to detect the amplified DNA from *Staphylococcus aureus* (*S. aureus*) and *Klebsiella pneumoniae*. One year later, they successfully detected a single cell of *Vibrio cholerae* in a pure water sample in just 20 min using particle diffusometry [103]. Their assay displayed 10-fold higher sensitivity than the gold-standard, real-time LAMP. On the other hand, Das et al. [104]. developed a Janus particle-enabled rotational diffusometric sensor paired with LAMP for *Escherichia coli* (*E. coli*) gDNA. Analysis of 300 images of blinking signals from 1 µm sized half-fluorescent half-gold functionalized Janus particles via a cross-correlation algorithm obtained a LOD of 48.2 pg/µL of *E. coli* gDNA in just 10 min, with a sample volume as low as 2 µL. In the follow-up article, the authors reported the detection of SARS-CoV-2 *nsp-2* cDNA in a recombinant plasmid with a LOD of 70 ag/µL in 10 min using rotational diffusometry combined with LAMP [105]. It 10-fold higher sensitivity compared to the gold standard: real-time PCR. In another strategy, a smartphone-based POC device was developed for end-point detection of LAMP amplicons using particle diffusometry. This device was used to detect *Vibrio cholerae* [106] and *Plasmodium falciparum* [107] with a LOD of 6 cells/reaction (0.66 aM) in 35 min and a LOD of 3 parasites/μL from a blood sample in 45 min, respectively. The device consists of a 3D-printed platform with a microscopic lens, an LED light, and a smartphone to take a video of the amplified sample with fluorescent beads. At a later date, Colbert et al. [108] modified this device further and reported the detection of SARS-CoV-2 using RT-LAMP. A portable heating unit and a microfluidic chip for performing RT-LAMP and sample analysis were further integrated into the device. This platform was capable of detecting 30 copies/µL of SARS-CoV-2 extracted RNA and 35 × 10^4^ virus copies/mL in diluted saliva. A comparison between various end-point detection methods of LAMP amplicons is summarized in Table 1.

### 3.2. Real-Time Detection

#### 3.2.1. Electrochemical Detection

**Figure 6:**
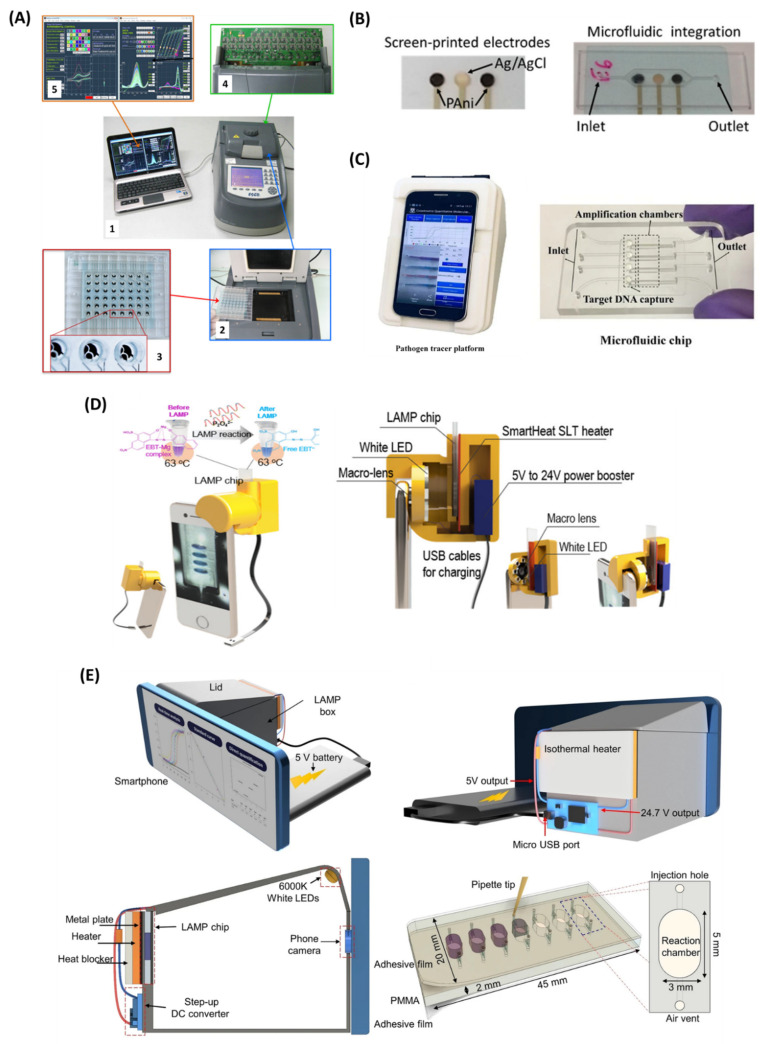
LAMP−based real-time detection modalities. (**A**,**A1**) Overview of the electrochemical readout device for real−time monitoring of LAMP reactions; (**A2**) PCR thermocycler attached with a flat Peltier-heating block with two 72-pin connectors; (**A3**) electrochemical microplate consisting 48 wells; (**A4**) PCB interface carrying the multiplexed potentiostat’s electronic circuitry; (**A5**) specialized software to extract the electrochemical response from each microwell and pilot the PCB interface. (**B**) Screen-printed pH-sensitive polyaniline (PAni)-based electrode for real-time detection of LAMP. (**C**) Hue-based detection of HPV and HIV by a “smart connected pathogen tracer” (SCPT) device. (**D**) Schematic illustration of smartphone-integrated i-Gene analyser and its inside view. (**E**) 3D schematics of i−Genbox for real-time detection of infectious pathogens. Reproduced with permission from references [109,110,116,118,119]. Copyright 2016, 2017, 2020 American Chemical Society, 2020 Springer Nature.

Real-time detection of LAMP products provides quantitative results without an additional sample preparation step. This saves time, limits the chances of false positives due to cross-contamination, and also delivers the advantage of extensive sample analysis with high accuracy. Thus, the LAMP-integrated real-time detection method can be used for the development of a portable, miniaturized POC diagnostic tool. The electrochemical detection method has been extensively used in real-time and quantitative analysis of LAMP amplicons (Table 2). Martin et al. [109] developed a LAMP-based electrochemical readout device for the detection of 48 samples in a real-time manner (Figure 6A). The device consists of a multiplexed potentiostat which enables simultaneous measurement of square-wave voltammetric responses for four different redox reporters, viz, [Os-(bpy)_2_dppz]^2+^, PhP, MB, and Ru(NH_3_)_6_^3+^. A LOD of as low as 2 copies/reaction was achieved for *Flavobacterium columnare* in just 30 min. In another work, Gosselin et al. [110] reported a screen-printed pH-sensitive polyaniline (PAni)-based electrode with an Ag/AgCl reference electrode for real-time detection of the LAMP assay of *Bacillus thuringiensis* with a LOD of 10 copies of DNA per reaction (Figure 6B). The PAni-based electrode detects the change in solution pH due to the polymerase enzyme activity during the LAMP reaction. A portable electronic acquisition card was used to obtain the potentiometric readouts, making this a suitable candidate for POC diagnostics of LAMP-based detection methods. Another LAMP-integrated, portable electrochemical device was developed for the detection of hepatitis B virus (HBV) DNA with a LOD of 6.18 fg/µL [111]. The drop-cell consisted of a platinum (Pt) working electrode connected to a potentiostat placed in a water bath. Methylene blue was used as a redox indicator to monitor the real-time electrochemical signals and the decrease in current due to the binding of MB with LAMP amplicons being measured by square-wave voltammetry (SWV). Similarly, Huang et al. [112] established a fully integrated electrochemical device combined with LAMP for the detection of *E. coli* O157:H7 and *S. enterica* targeting the *vt* and *invA* genes, respectively. The device was integrated with a gold electrode, and the redox molecule MB was used for electrochemical signal generation. The assay achieved a detection limit of LOD of 10 and 1 DNA copy per reaction in just 30 min for *E. coli* O157:H7 and *S. enterica*, respectively. This research group later developed a bis-intercalating redox reagent for real-time electrochemical qualitative analysis of LAMP amplicons. The redox probe was synthesized using two naphthoquinone-imidazole (NQIM) molecules which are covalently bonded by a linker group (R). This system achieved a LOD of single-copy *Salmonella* DNA in just 10 min [113].

#### 3.2.2. Smartphone-Based Real-Time Detection

It is an alternative method for the real-time monitoring of LAMP amplicons. This can be achieved by analysing the RGB value of the series of images captured during the amplification. The change in colour value is determined by using turbidity, colorimetric, and fluorescent dye. For example, Priye et al. [114] developed a portable LAMP device equipped with a smartphone for real-time detection of *Neisseria gonorrhoeae* (*N. gonorrhoeae*). This method utilizes the chromaticity-luminance methodology to measure the fluorescence intensity of the amplified LAMP product, which delivered a detection sensitivity of 3.5 copies/10 μL sample. Similarly, Wang et al. [115] developed an on-chip POC device integrated with LAMP for viable *S. typhimurium* detection using a smartphone-based real-time turbidity method. The *Salmonella* cells were mixed with propidium monoazide (PMA) and anti-*Salmonella* capture antibody-coated magnetic nanoparticles for the pre-treatment of dead bacterial DNA. The system was capable of detecting 14 CFU/mL of *S. typhimurium* in chicken meat within 90 min. Another smartphone-based quantitative molecular diagnostic platform was developed for the real-time colorimetric defection of LAMP assay [116] (Figure 6C). This low-cost and portable device was successful in the quantitative analysis of HPV DNA from saliva and vaginal swabs and HIV RNA from plasma samples. In another study, Thio et al. [117] developed a lab-on-a-smartphone (LOS) platform integrated with a plasmonic-enhanced optoelectrowetting (OEW) device for monitoring bacterial contamination in environmental water samples. The integration of the OEW enables pumpless droplet manipulation for on-chip sample preparation. It utilizes the smartphone camera as an optical detector for the colorimetric study through red–green–blue (RGB) analysis using the image processing application. This advanced device delivers real-time detection of *E. coli* in just 30 min. In another work, Nguyen et al. [118] developed a 3D-printed, portable smartphone-based integrated genetic unit (i-Gene) for the POC diagnostics of infectious pathogens. The LAMP amplification was performed on a microfluidic chip and the heater for LAMP was powered by the smartphone (Figure 6D). The smartphone was powered by a 5.0 V power bank. Eriochrome Black T (EBT) was used for colorimetric detection of the LAMP reaction. Upon gene amplification, the colour change of the sample was visualized in real-time by the CMOS sensor of the smartphone camera, enabling sensitive detection of *E. coli* O157:H7 with a LOD of 10 copies/µL in just 60 min. In the same year, they developed a smartphone application for the colorimetric detection of LAMP and measured the DNA copy number in the test sample using a calibration curve plotted to form the known template concentration vs. threshold time [119] (Figure 6E). This fully integrated smartphone-based device is an ultimate solution for the POC testing of infectious pathogens and early disease control, especially in developing countries and resource-limited situations. Two years later, this group developed an Internet of Things (IoT)-based portable, lightweight (320 g), and miniaturized diagnostic device for rapid COVID-19 screening [120]. This fully automated device was powered by a portable battery and operated with a smartphone. An integrated microfluidic chip was utilized for viral lysis, RNA extraction, and RT-LAMP reaction targeting multiple genes (N, E, and As1e genes) of SARS-CoV-2. RT-LAMP was monitored in real-time by measuring the fluorescence intensities via a CMOS sensor upon excitation with a 488 nm wavelength of LED light. The recorded data were processed and analysed by a microprocessor installed in the device and wirelessly transferred and displayed on the smartphone. A LOD of 20 copies/µL was achieved with an assay time of 65 min using SYBR green dye.

**Table 2:** LAMP-based real-time detection methods.

Technique	Pathogen	Signal Transduction Material	Detection Method	Time	LOD	Reference
Electrochemical	*Salmonella*	Bis-NQIM-R (naphthoquinone- imidazole)	Differential pulse voltammetry (DPV)	10 min	1 copy/reaction	[113]
	*Flavobacterium columnare*	[Os-(bpy)_2_dppz]^2+^, PhP and MB and Ru(NH_3_)_6_^3+^	Square wave voltammetry (SWV)	30 min	2 copies/reaction	[109]
	*E. coli O157:H7* *S. enterica*	Methylene blue	Differential pulse voltammetry (DPV)	30 min	10 copies/reaction 1 copy/reaction	[112]
	Hepatitis B virus	Methylene blue	Square wave voltammetry (SWV)	60 min	6.18 fg/µL	[111]
Smartphone-based	*E. coli O157:H7*	Eriochrome Black T (EBT)	Colorimetric	60 min	10 copies/μL	[118]
	*N. gonorrhoeae*	SYTO	Fluorescence	40 min	3.5 copies per 10 μL	[115]
	*SARS-CoV-2*	SYBR	Fluorescence	65 min	20 copies/ μL	[120]

## 4. LAMP-Based Point-of-Care Biosensors

### 4.1. LAMP-on-a-Chip

#### 4.1.1. Classical Microfluidic Chip

**Figure 7:**
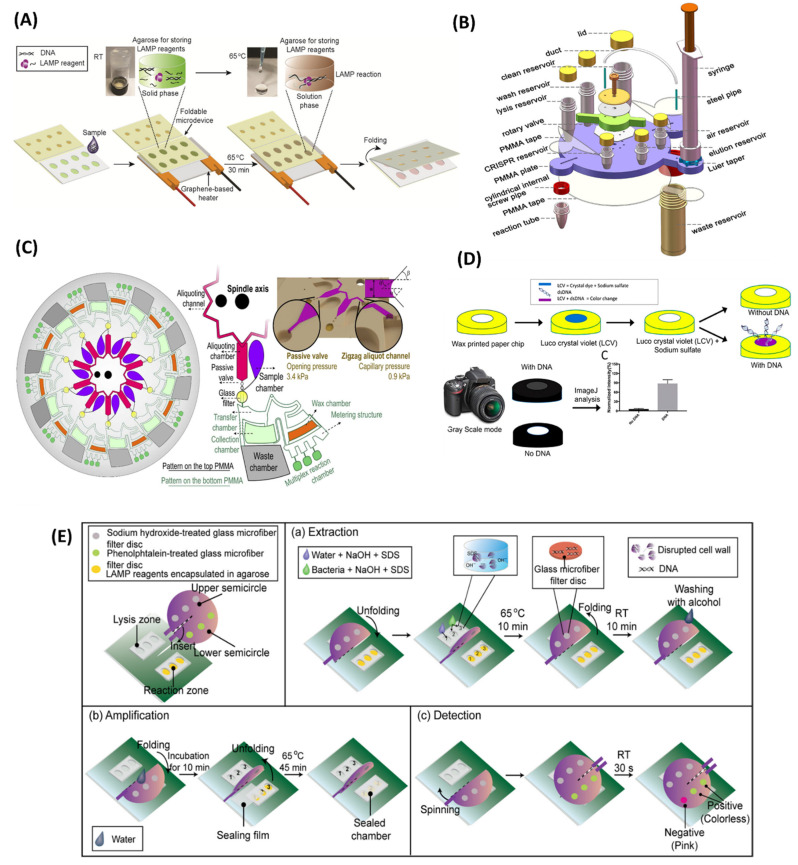

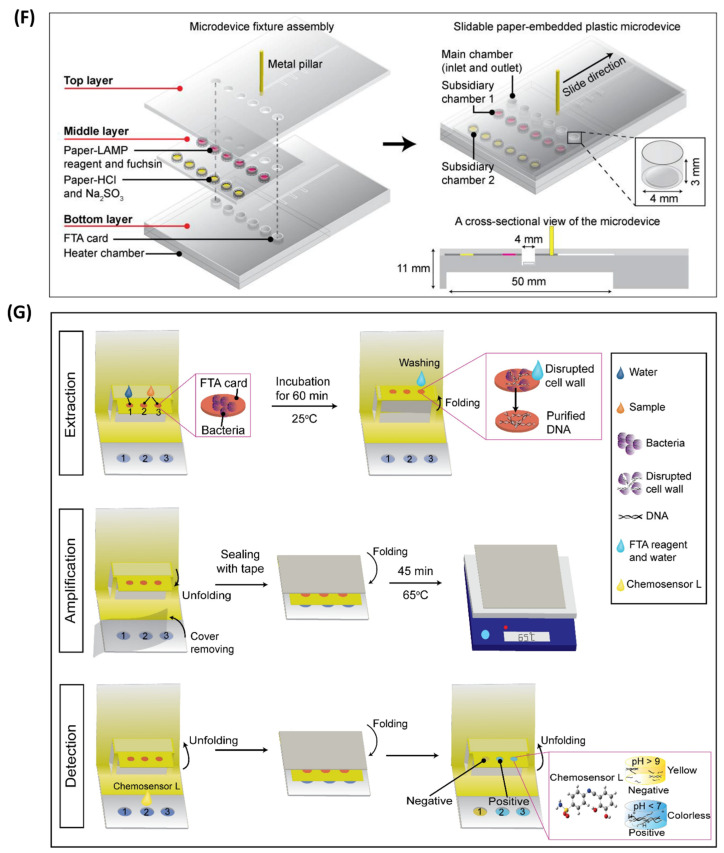
LAMP-on-a-chip biosensors. (**A**) Schematic workflow of graphene-heater-integrated polycarbonate-based foldable microdevice. (**B**) Structural overview of a rotary valve-assisted fluidic chip. (**C**) Centrifugal microfluidic disc for multiplex RT-LAMP. (**D**) Schematic workflow of colorimetric detection of LAMP amplicons on a wax-printed paper microdevice. (**E**) Working principle of paper-based microdevice with a spinning mechanism. (**F**) Structural overview of FTA card-based fully integrated slidable paper microdevice. (**G**) Illustration of a pop-up, paper-based, fully integrated microfluidic platform. Reproduced with permission from references [121,124,130,133,138,145,146]. Copyright 2017, 2019, 2021 American Chemical Society; and 2019, 2021, and 2022 Elsevier.

Controlling the spread of a contagious disease demands extensive rapid testing. This may lead to a risk of cross-contamination and further transmission of the infection. Additionally, the large amount of sample requirement makes the diagnosis process expensive and limits mass testing. The introduction of microfluidic devices can overcome this limitation and provide a “sample-in-result-out” route in a protected environment. Integration of LAMP with an advanced microfluidic chip further enhances the analysis and delivers rapid and highly specific detection with very high sensitivity. In recent years, LAMP-on-a-chip has emerged as an advanced solution for POC diagnostics for contagious diseases. Polymethyl methacrylate (PMMA) and poly-dimethylsiloxane (PDMS)-based classical microfluidic chips are the most common examples of LAMP-on-a-chip modalities. Here, we summarize the recent advances in LAMP-integrated microfluidic devices. Trinh et al. [121] developed a LAMP-based foldable microdevice for the detection of multiple foodborne pathogens. This device is made up of a polycarbonate thin film with two different sections for LAMP reaction and analyte detection (Figure 7A). The reaction part is enclosed with 2-hydroxyethyl agarose containing the LAMP reagents for their long-term storage and maintenance. It can successfully store the LAMP reagents for up to 45 days. On-chip amplification is performed by using a graphene-based heater. The assay achieved a LOD of 2.5 × 10^2^ copies/reaction in 30 min. In another study, Fu et al. [122] developed a LAMP assay using the most probable number (MNP) for the quantification of *E. coli* and *Enterococcus* spp. in a PMMA chip. The assay achieved a LOD of 4 copies/well in 35 min. A LAMP-integrated POC microdevice coupled with gold nanoparticles was developed by Sivakumar et al. [123] for the detection of *Enterococcus faecium* and SARS-CoV-2 plasmids. In the presence of LAMP product, the gold chloride is reduced to red-coloured gold nanoparticles under UV light, providing a detection limit of 42 fg/µL for COVID-19 plasmids in just 10 min. In another effort, a fully integrated rotary valve-assisted microfluidic device was developed for the DNA extraction, amplification, and detection of *V. parahaemolyticus* and spiked shrimp with a LOD of 3.1 × 10^1^ copies per reaction in 80 min [124] (Figure 7B). The CRISPR/Cas12a method was used for the detection of the LAMP amplicons. The fluorescence signal of the amplified product was recorded in real-time PCR. The detection cost per test was less than 4 USD. At a later time, a classical microfluidic device was developed by Tian et al. [125] for the detection of *Cryptococcal meningitis* (CM). The five-layer microfluidic module provided cellular lysis, DNA extraction, and purification for the isothermal amplification. LAMP was performed in a portable bead consisting of lyophilized reagents. This module can be utilized as an auxiliary tool for the middle and late phases of CM and to confirm instances with ambiguous lateral flow assay (LFA) results in the early stages of CM diagnosis.

Recently, microfluidic devices are also integrated with RT-LAMP for the detection of the highly infectious SARS-CoV-2 virus. For example, Mateos et al. [126] devised a PMMA-made, integrated microfluidic chip for magnetic bead-based RNA extraction and RT-LAMP reaction targeting ORF1a and N gene of SARS-CoV-2. The microchip contains a large chamber for sample loading, followed by seven wash chambers and a detection chamber. The use of a greater number of wash chambers delivers high-purity RNA extraction and eliminates carryover contaminations. This device achieved a detection sensitivity of 470 copies/mL with a turn-around time of 60 min. This simple-to-use platform delivers rapid and accurate detection of SARS-CoV-2 and could be used for mass screening of COVID-19 at POC. Similarly, Jhou et al. [127] designed an RT-LAMP-based integrated microfluidic platform (IMP) for real-time detection of the SARS-CoV-2 virus. This automated platform consists of a PDMS-based microfluidic chip for viral lysis and RNA extraction, and a heating module for RT-LAMP. Liquid samples were transported inside the chip via micropumps and valves by creating a gauge pressure difference, controlled by a fluidic control module, and also functioned as micromixers for mixing the reagents. The calcein-based fluorescent detection obtained a LOD of 5 × 10^3^ copies of viral RNA per reaction in 90 min.

The LAMP-on-a-chip system also provides the advantage of multiplex detection, maintaining the virtue of LAMP. For example, Liu et al. [128] developed a fully automated centrifugal chip for the simultaneous detection of five different bacteria. The automated design provided the bacterial cell lysis, DNA purification, LAMP reaction, and the end point colorimetric detection of the samples with a LOD of 10 copies/µL in just 70 min. Bead-beating-based mechanical lysis was used to substitute the chemical lysis. A colour sensor was used with a white LED light for on-chip LAMP readouts. Meanwhile, a disc-shaped dual-sample microfluidic chip made out of PMMA was developed by Jin et al. [129] for multiplex detection of 10 waterborne bacteria. The microfluidic disc was equipped with a centrifugal module that enables the reagent to flow through the channel. The real-time fluorescence resulted from a LOD ranging from 7.92 × 10^−3^ to 9.54 × 10^−1^ pg per reaction in 35 min, demonstrating high specificity and reproducibility. In another study, a centrifugal chip-based, fully integrated portable genetic analyser was developed for multiplex RT-LAMP [130]. The centrifugal chip allowed the detection of 10 samples in one run. The chip consisted of 10 aliquoting chambers which were connected with zigzag aliquoting channels and a glass filter column for RNA extraction. The structure of the disc is shown in Figure 7C. A passive valve was used between the aliquoting chamber and the glass filter column to manipulate the fluid flow. Capillary pressure enabled the automatic sample distribution in aliquoting chambers, and the subsequent washing and elution of samples were achieved by centrifugal protocols. Each sample had three reaction chambers on the chip, preloaded with freeze-dried LAMP primers for simultaneous amplification of ORF1ab, N, and S genes. To prevent sample evaporation, the reaction chamber was blocked by wax. The fluorescence signals of the amplified products were recorded, and the three target genes of SARS-CoV-2 in 10 samples were detected with a LOD of 20 copies/µL with a turnaround time of 90 min.

#### 4.1.2. Paper-Based Chips

Conventional microfluidic devices often require additional pumping and micro-valves for fluid movements. This increases their operational complexity, use of raw materials, and overall cost. However, paper-based and paper–polymer hybrid microfluidic devices have emerged as an alternative solution, due to their high biocompatibility with nucleic acids and proteins, ease of fabrication, and low production cost [131]. Additionally, the capillary force can eliminate the use of micropumps and other bulky equipment. Rodriguez et al. developed a low-cost, sample-to-answer paper-based molecular diagnostic assay integrated with RT-LAMP for the detection of H1N1 from human nasopharyngeal swabs with high sensitivity. The RNA extraction from the clinical samples and purification, followed by the RT-LAMP reaction, was performed in the paper device. Naked eye detection on lateral flow strips delivered a LOD of 10^6^ copies/mL within just 45 min, which is 10-fold more sensitive than the current immunoassay technique [132]. In another work. a cellulose-based paper microchip was developed by Roy et al. [133] for the detection of microbes in porcine meat (Figure 7D). Amplified LAMP product was detected in a wax-printed cellulose paper-based microchip using crystal violet dye. This low-cost colorimetric assay delivered rapid and highly sensitive detection of *Bacillus subtilis* and *Sus scrofa* with LODs of 10 pg/μL (2.2 × 10^3^ copies/μL) and 1 pg/μL (3.43 × 10 ^−1^ copies/μL), respectively. Similarly, Kaarj et al. [134] designed a wax-printed G4 cellulose paper chip in combination with RT-LAMP for POC diagnosis of Zika virus from water, urine, and diluted blood plasma. The paper chip was divided into two parts: the sample-loading region and the detection/amplification area. The sample containing the spiked viral particles was transferred into the loading region, and the sample moved through the channel via capillary action. The detection area was placed on a hot plate, and the RT-LAMP reagents were added. A LOD as low as 1 copy/µL was achieved with smartphone-based colorimetric detection in 15 min from the biological samples. In another effort, Batule et al. [135] demonstrated a two-step strategy for viral RNA extraction, followed by isothermal amplification and detection in a paper substrate. In the first step, a nitrocellulose-based paper strip was used for the extraction of viral RNA from serum within 5 min. In the next step, RT-LAMP was performed in the paper chip consisting of four reaction chambers connected via a fluidic channel and pre-loaded with reaction pads. The RT-LAMP reagents, including the reverse transcriptase, primers, *Bst* polymerase, and the dye for visual detection, were dry stored in the reaction pad. The assay achieved detection limits of 1 copy and 10 copies of three different RNA particles (e.g., zika, chikungunya, and dengue) in phosphate buffer solution (PBS) and serum, respectively. The whole process of RNA extraction and detection was completed within 1 h.

Paper-polymer-based hybrid microfluidic devices also obtained high attention and were applied in the detection of microbial infections. Pang et al. [136] reported a hybrid microfluidic platform based on PDMS/paper for the detection of food-borne pathogens. The microdevice contained three LAMP reaction chambers connected by microchannels, including one for a negative control. The paper disc was pre-loaded with LAMP reagents and placed in the reaction chambers. The high gas solubility of PDMS enables the self-priming of samples. The use of mixed dye delivered visual detection of the final product with a LOD of 10^3^ CFU/mL for *S. aureus* and *V. parahaemolyticus*. Similarly, Trinh et al. [137] developed a paper-embedded foldable microdevice for naked-eye detection of *Salmonella* spp. with a LOD of 10^2^ CFU/mL from milk samples in 65 min. The microdevice consisting of three sections attached with a sealing film was able to perform nucleic acid extraction, LAMP reaction, and colorimetric detection. The sample and reaction chambers were placed up of polycarbonate consisting of nine chambers, pre-loaded with LAMP reagent cellulose paper. The fuchsin-coated paper strip was used in the detection layer, where it changed colour to purple in cases of positive LAMP. Another LAMP-integrated paper-based microdevice was reported with a spinning mechanism for the detection of vancomycin-resistant *Enterococcus* (VRE) [138]. The stationary part consisted of a PDMS chip for LAMP amplification. The spinning part was installed with a three-glass microfiber treated with sodium hydroxide for DNA extraction and another three-glass microfiber treated with phenolphthalein for the colorimetric detection of the LAMP amplicons (Figure 7E). This device was capable of detecting 10^2^ CFU/mL of VRE in tap water in 45 min. In another strategy, a foldable paper microdevice was developed by Dinh et al. [139] to perform on-chip LAMP amplification. The polymerized Safranin O interacts with LAMP amplicons, resulting in a dark red solution. Limits of detection of 10^−4^ ng/µL and 10^2^ CFU/mL were achieved for SARS-CoV-2 plasmid and *E. faecium* in just 60 min. In a similar approach, a paper-based microfluidic chip was developed by Zhang et al. [140] for colorimetric detection of foodborne bacteria *E. coli* O157:H7. This microchip was further utilized for on-chip detection of *Salmonella* spp. from milk samples. Bacterial cell lysis was executed through thermal shock at 90 °C for 5 min. The detection was performed by calcein dye embedded in the reaction chamber, delivering LODs of 13.4 pg/µL and 12 CFU/mL for *E. coli* O157:H7 and *Salmonella* spp., respectively. To summarize, the paper-based tests meet the criteria for POC diagnostic tools and have the potential to be used in the early and onsite diagnosis of infectious diseases.

#### 4.1.3. Fully Integrated Chips

Microfluidics chips integrated with nucleic acid extraction, purification, and LAMP amplification are suitable candidates for on-site detection, especially in resource-limited regions. In past years, researchers have attempted to develop microdevices integrated with sample pre-treatment functionalities, including the isothermal amplification in a single microfluidic device. Materials such as a magnetic bead, silica bead, glass filter membrane, and FTA card were used for nucleic acid extraction and purification. Guo et al. [141] designed a microfluidic chip integrated with silica-based solid-phase extraction (SPE) of bacterial genomic DNA and LAMP amplification for the detection of three different bacteria, including *E. coli* O157:H7, MRSA, and methicillin-sensitive *Staphylococcus aureus* (MSSA). The microchip consisted of two channels: one for sample loading, and the other one for performing the washing step. Both channels were loaded with a micro-valve to control the fluid flow. The extracted genomic DNA was only allowed to pass through the SPE chamber, and the silica beads were blocked in the micro-pillars. A detection limit of 10^2^ CFU/100 µL was achieved for three specific genes, *rfb*E, *spa*, and *mec*A, with a turnaround time of 2 h. Another LAMP-based, nucleic-acid-extraction-integrated lab-on-a-disc platform was developed by Loo et al. [142] to deliver a sample-to-result solution. A silica membrane was used for on-chip solid-phase extraction and purification of the bacterial DNA. The fluid flow in the microfluidic disc was controlled by the centrifugation force. Real-time fluorescence monitoring employed by SYTO-9 fluorescent dye provided a detection limit of 10^3^ CFU/mL for *Mycobacterium tuberculosis* in sputum and 10^2^ CFU/mL of *Acinetobacter baumanii* (Ab) in blood. The entire process can be performed by this microfluidic platform in less than 120 min, and multiple samples can be analysed simultaneously. Similarly, Park et al. [143] devised a compact-disc, rotary microfluidic device integrating nucleic acid extraction based on glass microbeads, chambers for LAMP reaction, and three separate lateral flows strips for simultaneous detection of multiple food-borne pathogens in contaminated milk or water samples. The glass microbeads were loaded in the microchannels where the genomic DNA was adsorbed on silica via hydrogen bonding, in the presence of chaotropic salts. DNA extraction in the microfluidic disc was confirmed by using a FAM-labelled artificial DNA probe. Buffer transport was achieved by the centrifugal speed, and the sample flow was controlled with the help of multiple siphons and a circular capillary valve. Target genes of *S. typhimurium* and *V. parahaemolyticus* were simultaneously detected with a LOD of 50 CFU with an assay time of 80 min. Although these integrated microfluidic discs are capable of fully automated, rapid, and sensitive detection of pathogens in a multiplex manner, the treatment of large sample volumes is still a major drawback. To solve that problem, an integrated centrifugal microfluidic chip was designed for LAMP-based multiplex detection of food-borne bacteria with a sample volume of up to 1 mL. The microdevice consisted of two major parts: a 3D-printed cartridge for LAMP reagent storage and a centrifugal disc to release the solutions into the microdevice, which is controlled by disc rotation. To absorb the washing solution and the extra sample, a super-absorbent polymer (SAP) was installed in the washing chamber, which eventually allowed the use of samples up to 1 mL. Twenty reaction chambers were used in each part of the chip, enabling simultaneous detection of multiple samples in one run. Bead-based DNA extraction allowed the full automation of the process, and the colorimetric detection of three bacteria (*E. coli* O157:H7, *S. typhimurium*, and *V. parahaemolyticus*) was performed within 1 h with a LOD of 10^2^ cells/mL [144]. A Flinders Technology Associates (FTA) card-embedded sliding hybrid microfluidic device was developed, consisting of three layers, to perform the DNA extraction, LAMP reaction, and bacterial detection simultaneously (Figure 7F). An FTA card was used for bacterial DNA extraction and purification. Colorimetric analysis was performed using a fuchsin-based method which eventually detects *Salmonella* spp. and *E. coli* O157:H7 with a LOD of 30 CFU/sample and *S. aureus* with a LOD of 3 × 10^2^ CFU/sample in 75 min [145]. Similarly, a 3D-paper-based microfluidic device inspired by a pop-up greeting card was developed for the detection of VRE [146]. The FTA card was installed on the top for the DNA extraction, and the PDMS chip served as the LAMP reaction chamber (Figure 7G). The paper was coated with concentrated nail polish to prevent the sample flow throughout the paper by modifying the porous structure. A pH-sensitive chemosensor delivered a detection of VRE with a LOD of 10^2^ CFU/mL from raw milk samples in 45 min. Another microfluidic platform was designed by Tsai et al. [147] and integrated with an electromagnet for viral lysis, RNA extraction, and detection of SARS-CoV-2 by RT-LAMP in real time. The electromagnets enabled the bead-based RNA extraction and the mixing of the reagents. The assay achieved a detection limit of 5000 viral copies per reaction within 82 min. Table 3 listed the recently developed LAMP-on-a-chip modalities and provides a comparison between them.

**Table 3:** Summary of LAMP-based microfluidic platforms.

Technique	Target	Pathogen	Material	Readout	Time	LOD	Reference
Classical	DNA	*Salmonella* spp. *E. coli* O157:H7	Polycarbonate	Colorimetric	30 min	2.5 × 10^2^ copies/mL	[121]
	DNA	*E. coli* and *Enterococcus* spp.	PMMA	Colorimetric	35 min	4 copies/well	[122]
	DNA	*V. parahaemolyticus*	PMMA	Fluorescence	80 min	3.1 × 10^1^ copies/reaction	[124]
	RNA	SARS-CoV-2	PMMA	Fluorescence	90 min	20 copies/µL	[130]
Paper-based	DNA	*Sus scrofa* (porcine) *Bacillus subtilis*	Cellulose paper	Colorimetric	10 min 18 min	3.43 × 10 ^−1^ copies/μL 2.2 × 10^3^ copies/μL	[133]
	DNA	*Salmonella* spp.	Polydopamine coated paper-polycarbonate	Colorimetric	65 min	1 × 10^2^ CFU/mL	[137]
	DNA	VRE	Paper-PDMS	Colorimetric	45 min	10^2^ CFU/mL	[138]
	RNA	Zika virus	G4-cellulose paper	Colorimetric	15 min	1 copy/µL	[134]
Integrated chips	DNA	*E. coli* O157:H7, MRSA, MSSA	Silica beads-PMMA	Fluorescence	2 h	10^2^ CFU/100 µL	[141]
	DNA	*S. typhimurium* and *V. parahaemolyticus*	Glass microbeads-PPMA	Colorimetric	80 min	50 CFU	[143]
	DNA	VRE	FTA card-PDMS	Colorimetric	45 min	10^2^ CFU/mL	[146]
	RNA	SARS-CoV-2	Magnetic beads	Fluorescence	82 min	5000 copies/reaction	[147]

### 4.2. Digital LAMP

**Figure 8:**
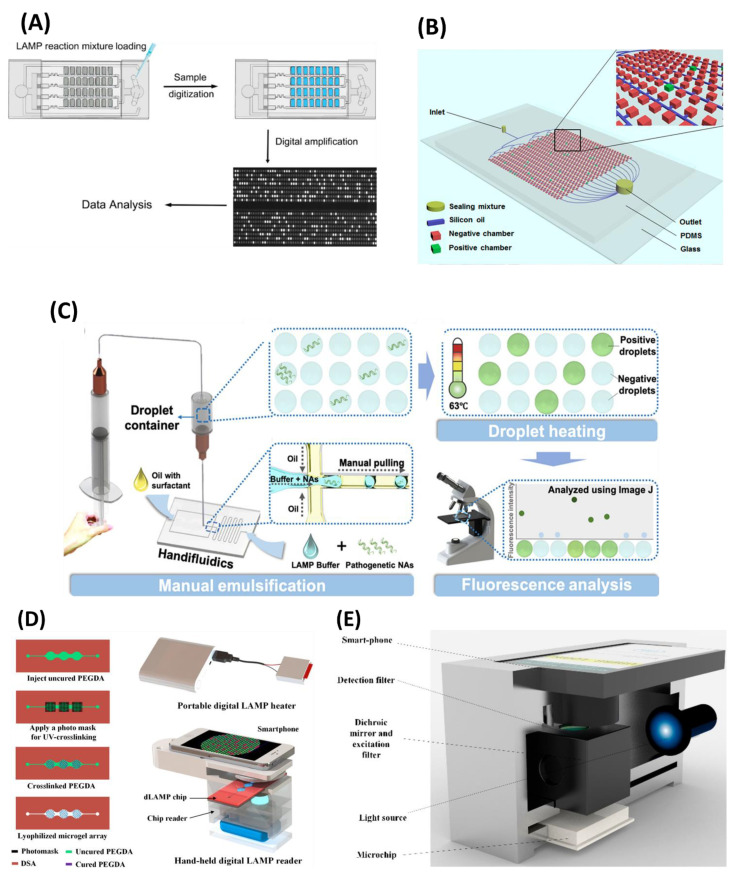
Schematic illustration of digital LAMP methods for detection of LAMP amplicons. (**A**) Self-digitization dLAMP chip. (**B**) Self-compartmentalization microfluidic chip for digital detection of VP. (**C**) Handifluidics ddLAMP device integrated with LAMP mixture emulsification. (**D**) Microscale hydrogel chip integrated dLAMP device. (**E**) Smartphone imaging-based dLAMP device. Reproduced with permission from references [149,151,154,155,156]. Copyright 2017, 2020, and 2021 American Chemical Society.

In recent years, digital LAMP (dLAMP) has gained popularity for the accurate measurement of nucleic acids within a sample. Based on the sample separation, dLAMP can be categorized into two groups: droplet-based digital LAMP (ddLAMP) and well-plate-based dLAMP. Both methods rely on separating the sample into thousands of nanolitre-sized droplets containing one target molecule each. The sample droplets can be created by suspending them in oil or loading the sample into a microwell and sealing with oil or a lid. The number of target molecules can be quantified by analysing the number of droplets that display a positive signal. Amplification results are analysed by digital image processing or flow cytometry. Gansen et al. [148] reported the first sample self-digitization (SD) chip-based digital LAMP (dLAMP) for amplification and detection of a single DNA/RNA molecule in a stationary droplet. The chip was replicated in PDMS containing 5000 microwells. Droplets were created by mixing oil and the sample through air pressure, and the LAMP was performed at 65 °C. The device showed adequate size homogeneity and low sample consumption. Using this concept, Wang et al. [149] reported the detection of 14 high-risk HVP in clinical samples with very high specificity and accuracy (Figure 8A). Alternatively, Zhu et al. [150] developed a self-priming compartmentalization (SPC) chip to perform digital LAMP analysis. The vacuum generated via degassing the air-dissolved PDMS chip provided the pumping force for the sample and oil flow in the chip. This allowed the droplet creation and self-compartmentalization of the sample without using microvalves and other control systems. A LOD of 1 × 10^3^ CFU/mL for *V. parahaemolyticus* in contaminated food samples was achieved with this device [151] (Figure 8B). However, long-term preservation of a vacuum may not be possible, which will impede self-priming. To overcome this problem, Ma et al. [152] proposed a hydrophilic film-coated self-driven PDMS chip to conduct dLAMP. The hydrophilic film enables automatic sample manipulation in the chip via capillary forces. Under such conditions, the assay achieved the LOD of 11 copies per 30 µL sample for VRE within 30 min of the LAMP reaction. Meanwhile, a droplet-based microfluidic device for dLAMP was developed by Rane et al. [153] for the detection of *N. gonorrhoeae*. A continuous flow of oil and the sample through the cross microchannel generated 100,000 droplets per 110 min with a size of 10 picolitres, containing the single nucleic acid and LAMP reagent (Table 4). These droplets were moved toward the heating region for LAMP amplification. Calcein dye-based fluorescent detection using confocal fluorescent spectroscopy delivered a LOD of 600 copies/µL.

Droplet-based digital LAMP requires a highly trained workforce with experience in microfluidics and sophisticated instruments. Therefore, to make ddLAMP more convenient, Yuan et al. [154] designed a “Handifluidics” device integrating the LAMP mixture emulsification, incubation region, and fluorescence detection of the amplified product (Figure 8C). A manually operated syringe was used to generate monodispersed droplets in a PDMS chip. The emulsified LAMP droplets were collected in the droplet container and heated in the water bath at 63 °C to perform the LAMP. Fluorescent microscopy was used to quantify the target with a LOD of 10 DNA copies/µL. In another effort, Cao et al. [155] developed a microscale, hydrogel-chip-integrated, miniaturized dLAMP device for the detection of λDNA templates with a LOD of 1 copy/µL. The chip contains more than 10,000 microgels capable of self-partitioning the DNA molecules (Figure 8D). The platform was integrated with a heater for LAMP reaction, and the image analysis was performed using a smartphone-based digital readout. Similarly, Hu et al. [156] created a portable sample-to-result digital LAMP platform that incorporated nucleic acid extraction and smartphone imaging-based fluorescence detection (Figure 8E). Surface-tension-assisted immiscible phase filtration (IFAST) was used to extract nucleic acids. Negative pressure mixing and sample partitioning were achieved using a spiral mixing channel and a flow-focusing droplet production mechanism. This device was capable of detecting low-abundance cfDNA and EGFR L858R mutation. The suggested device can provide a low-cost, simply produced, and user-friendly solution for POC tests of nucleic acid in resource-limited situations.

“SlipChip” is another microfluidic platform where two plates are connected [157]. The bottom layer contains an array of microchambers and ducts. The top layer consists of wells and acts as a cover. The sample is loaded in the top-layer wells through microchannels and mixed with the preloaded reagents in the bottom chambers by slipping the top plate. This platform eliminates the use of pumps and valves and also enables multiplex detection with a sample volume in pico/nanolitres. Through a similar approach, Sun et al. [158] reported a SlipChip device for the detection of HIV RNA by performing digital two-step RT-LAMP. First, single RNA molecules were compartmentalized using Poisson distribution and the corresponding cDNA was synthesized. Later, those cDNAs were subjected to LAMP amplification. This two-step dRT-LAMP obtained 10-fold higher efficiency compared to the single-step RT-LAMP. Similarly, a self-partitioning SlipChip (sp-SlipChip) platform was developed for the quantification of HPV in clinical samples. The chip utilizes the capillary pressure to undergo self-partition for independent droplet creation. The reagent was loaded into a “chain-of-pearls”-shaped channel in the bottom plate. A salinized hydrophobic top plate containing the expansion channel is moved by manual slipping and aligned with the bottom channels. The difference in capillary pressure breaks down the fluid and self-partitions it into individual droplets [159]. Therefore, a precise alignment of the microchannels can be eliminated, making this device a promising point-of-care diagnostics tool for infectious pathogens. In conclusion, including dLAMP in a sample-to-answer microfluidic device has a lot of promise for achieving speedy and sensitive detection.

**Table 4:** Comparisons of various digital LAMP modalities.

Target	Pathogen	Reaction Chamber Type	No. of Chambers	Sample Volume	LOD	References
DNA	*Streptococcus pneumoniae*	Droplet	6500	~2.3 nL	10 copies/μL	[154]
λDNA	EGFR L858R mutation	Droplet	10,000	~1 nL	1 copy/μL	[155]
DNA	*N. gonorrhoeae*	Droplet	100,000	~10 pL	600 copies/µL	[153]
Plasmid	HPV	Microwells	4480	~4.5 nL	1 fg/μL	[159]
DNA	VRE	Microwells	736	~22.6 nL	11 copies/30 μL	[152]
DNA	Β-lactin	Microwells	384	~6 nL	--	[150]

## 5. Application of LAMP in Clinical Diagnosis

Access to high-quality medical care and technologies is still a major issue in resource-constrained places, particularly in low- and middle-income countries. Therefore, it is crucial to develop POC devices that offer affordable, trustworthy, and quick screening of medical diseases outside of the laboratory setting. The World Health Organization (WHO) has established standards for assessing POC diagnostic platforms, with an acronym “ASSURED”—meaning they should have the following qualities: being low-cost/affordable, highly sensitive and specific (very low false positive), user-friendly (anyone can use), rapid/robust, high-tech equipment-free, and deliverable (portable). Due to its operational simplicity, LAMP is one of the best candidates to fulfil these criteria. Therefore, LAMP has been massively used in molecular diagnostics for various infectious diseases, especially during the time of COVID-19. For example, Song et al. [160] developed an instrument-free, two-stage isothermal amplification method called Penn-RAMP, combining reverse-transcription recombinase polymerase amplification (RT-RPA) and LAMP for the detection of SARS-CoV-2, targeting ORF1ab gene with improved efficiency. In this method, the RT-RPA is performed first in the cap of a reaction tube. The reaction was carried out at 38 °C with nasopharyngeal swabs and saliva samples. F3 and B3 LAMP primers and a recombinase polymerase enzyme were used for the RPA reaction. After 15–20 min, the RPA reaction mixture was transferred to the pre-loaded LAMP mixture in the same reaction tube. In the second stage, the LAMP reaction was performed at 63 °C for 40 min. RPA and LAMP mixtures were maintained in a 1:9 ratio to prevent LAMP inhibition due to RPA contents. The assay provided a LOD of 5 copies/reaction with a 10-fold higher sensitivity than RT-LAMP. Hence, Penn-RAMP overcomes the individual limitations of RPA and LAMP and delivers higher sensitivity. Additionally, integration of the CRISPR/Cas diagnostic method with LAMP provides the detection of pathogenic nucleic acids with high sensitivity and specificity. For example, Joung et al. [161] have developed STOPCovid (SHERLOCK testing in one-pot for COVID-19), a CRISPR/Cas-based one-pot detection method of SARS-CoV-2 combined with RT-LAMP. In this method, first, the viral RNA is isothermally amplified; then the amplicons are cleaved by a CRISPR-mediated reporter and sequentially detected. Viral RNA is extracted by thermal lysis at 60 °C for 10 min in a commercial lysis buffer. Thus, this method eliminates the additional viral RNA extraction step. After a 60 min reaction, the final product is identified by lateral flow strip or fluorescent reporter. The assay achieved a LOD of 100 copies/reaction in just 70 min. It also delivered very high accuracy (11/12) for clinical samples. Similarly, Broughton et al. [162] reported a rapid RT-LAMP-based SARS-CoV-2 detection platform named DETECTR (SARS-CoV-2 DNA Endonuclease-Targeted CRISPR Trans Reporter) with integrated CRISPR/Cas12 detection. This assay performs the RT-LAMP targeting of N (nucleoprotein) and E (envelop) genes of SARS-CoV-2, followed by Cas12 recognition of predetermined viral RNA sequences and virus detection on a lateral flow strip upon cleavage of the reporter molecule. A LOD of 10 copies/µL was achieved in just 40 min. Clinical samples were tested by extracting RNA from oropharyngeal/nasopharyngeal swabs of COVID-19 patients. The DETECTR assay showed 95% accuracy relative to the gold standard, RT-PCR.

### FDA-Approved LAMP-Based Devices

Various LAMP-based commercial POC test kits for SARS-CoV-2 were developed and received emergency use authorization (EUA) from the U.S. Food and Drug Administration (FDA). The Lucira COVID-19 All-in-one Test Kit was developed by Lucira Health Inc. [163] and received the EUA on 17 November 2020 for prescription domestic COVID-19 testing [164], and on 9 April 2021, the Lucira Check-it COVID-19 Test received authorization by the FDA for non-prescription home use [165]. It utilizes the RT-LAMP by targeting the N-gene of SARS-CoV-2 RNA. The colorimetric detection based on the pH change of the sample due to LAMP delivers the detection within 30 min. The nasal swab is dissolved in the elution buffer, which provides the viral lysis at room temperature. The buffer solution dissolves the preloaded lyophilized reagents, and the colorimetric readout is delivered by an electronic and optical element in the test kit. No pumps are needed because the device uses capillary flow and gravity, and it just has one user-activated valve. The heater and optics are located on a single PCB. A LOD of 2700 copies/swab was achieved for Lucira Check-It, with a detection accuracy of 98%. Similarly, Detect Inc. combined RT-LAMP with lateral flow technology and developed the Detect COVID-19 test kit [166]. This test kit targets the ORF1ab gene of the SARS-CoV-2 viral genome. The Detect Hub is used in conjunction with the disposable test tube and collection buffer. After connecting, the Detect Hub must be kept aside for 65 min before the test can be conducted, likely to stabilize the system. The RT-LAMP reaction will begin automatically when the tube has been inserted into the Detect Hub. The amplification takes about 55 min, followed by a lateral flow assay for 10 min, and the read-out of the result is performed using the Detect app on a smartphone. The kit provides a human positive control to confirm the appropriate sample collection and the viral RNA extraction. Although the Detect COVID-19 test is intended to be used at home, only healthcare professionals could purchase IFU. On 11 April 2022, the FDA issued a letter of authorization to Detect Inc. [167]. The test may be used at home without a prescription. Table 5 listed the recently developed LAMP-based molecular diagnostic methods for SARS-CoV-2 detection and provides a comparison between them.

**Table 5:** RT-LAMP-based methods for COVID-19 diagnosis.

Method	Target Gene	Time	Sensitivity	Specimen (Swab Type)	LOD	EUA by FDA	Price Per Unit
STOP Covid [161]	N gene	70 min	91.6%	Nasopharyngeal and oropharyngeal swabs	100 copies/reaction	No	$40 USD
Penn-RAMP [160]	ORF1ab and N gene	60 min	84%	Nasopharyngeal swab and saliva	5 copies/reaction	No	NA
DETECTR [162]	N and E gene	40 min	95%	Nasopharyngeal and oropharyngeal swabs	10 copies/µL	No	NA
iSCAN [168]	N and E gene	60 min	86%	Nasopharyngeal swab	10 copies/sample	No	$2–5 USD
iLACO [169]	ORF1ab gene	40 min	89.9%	--	10 copies/µL	No	NA
Lucira Check-it [165]	N gene	30 min	98%	Nasal swab	2700 copies/swab	Yes	$68 USD
Detect COVID-19 test [166]	ORF1ab gene	65 min	95%	Nasal swab	800 copies/mL	Yes	$55 USD
Metrix COVID-19 test [170]	ORF1ab and N gene	30 min	95%	Nasal swabs and saliva	667 copies/mL	Yes	NA
DxLab COVID-19 test [171]	M gene	25 min	95%	Nasal swab	3000 copies/swab	Yes	NA

## 6. Limitations of LAMP

Despite its numerous advantages, LAMP exhibits several flaws that need to be fixed. In comparison with conventional PCR, LAMP exhibits less versatility in molecular biological applications. For example, LAMP produces a large-sized DNA chain, which impedes its utility in cloning and other biological applications [172]. Furthermore, the final product of LAMP is cauliflower-like DNA in diverse sizes, leading to the formation of smears or multiple banding on the gel. However, in the PCR, the final product appeared to be a single band. Hence, specific product identification on the gel is not possible in LAMP [173]. Other limitations of LAMP are discussed in this section.

### 6.1. Cross-Interference in Multiplex Detection

Multiplex detection in LAMP is still challenging due to its complex operational design, the specific primer requirement for every particular target sequence, and the formation of amplicons with multiple sizes. Conventional methods for enabling multiplex LAMP (mLAMP) involve adding an endonuclease recognition site to the LAMP primers, and then the digested amplicons are identified based on their sizes. However, the inadequate digestions of the amplified products produce multiple bands in the gel electrophoresis for each target, making multiplex LAMP detection challenging [174]. Other methods developed for mLAMP detection use molecular barcoding [175] and aptamer-modified gold nanoparticles [176]. However, the post-product detection processes are time-consuming, and the use of expensiveness of the sequencing tools and reagents increases the overall operation cost and prevents their use in wide applications. In addition, the post-amplification processing comprises a high risk of carry-over contaminations [39].

In real-time PCR, the DNA intercalating dye (such as SYBR Green) is used for the detection of a specific target gene sequence in a multiplex manner, and the amplified product is distinguished through melting curve analysis. However, the melting curve analysis cannot be used with LAMP due to the formation of products with multiple structures. Additionally, the strand displacement principle for DNA synthesis disables the use of probe-based detection. Furthermore, LAMP uses three primer pairs for each target, resulting in competition between the primers for *Bst* polymerase and other substrates, including dNTPs, MgSO_4,_ etc. Thus, optimizing the reaction conditions in mLAMP is challenging. However, designing a microfluidic chip with multiple reaction chambers should be able to overcome the above-mentioned limitations and provide detection of LAMP in a multiplex manner, by putting primers in various reaction wells for different targets. Microfluidic discs and paper chips are the perfect candidates for mLAMP.

### 6.2. Uncertainty of Primer Design

LAMP uses 4–6 primers that target 6–8 distinct regions in the small target segment with a distance of 120–160 bases between F2 and B2 and 40–60 bases between 5′ ends of F1 and F2 [11]. Therefore, primer design for specific targets with short sequences and a high number of mutations, especially in the case of RNA viruses, is still challenging. Primer Explorer V5 is the most commonly used online software for LAMP primer design. However, in certain cases, online primer design software is exposed to various restrictions and is unable to select the desired target sites, making primer design complicated. Hence, the primer design has to be performed manually. The position of the loop primers is also critical in primer design. The loop primers must be placed precisely between the B2–B1c and the F2–F1c sites, and should be oriented in a specific direction [18]. These substantial limitations on their arrangement disable the loop primer generation in some instances. Thus, different primer sets have to be chosen for loop primer generation. In addition, to find out the optimum performing primer for a particular sequence, multiple designs have to be selected, and the efficiency of the primer needs to be checked by trial and error. Furthermore, the use of large size (30–40 bases for FIP and BIP) and multiple primers elevates the chances of self-hybridization of the primers. This leads to the formation of a self-amplified product without any template, providing false-positive results [177]. In such cases, a redesign of the primer is recommended.

### 6.3. Carry-Over Contamination

Carry-over contaminations are a major drawback in LAMP due to the high volatility and product stability. Hence, unintended false-positive results are obtained in non-template controls [39,178,179,180]. Due to its high sensitivity, the LAMP reaction may produce false-positive results, even if only a little of the DNA from the amplification product is present. To prevent such cross-contamination, the use of laminar airflow for LAMP sample preparation and separate pipettes and filter tips are highly recommended. In addition, the opening of the reaction tubes containing the LAMP product and the post-amplification processes should be performed in an area isolated from the reagent preparation place. The reaction tubes should be closed immediately after the LAMP detection and disposed of in sealed double plastic bags. Autoclaving of the reaction tubes should be avoided to prevent the dispersion of the amplified product [39].

## 7. Conclusions and Perspectives

LAMP is a robust, powerful, and unique nucleic acid amplification technique that utilizes the strand displacement activity in an isothermal setting, thereby improving the limitations of PCR by eliminating the complex thermal cycling process. Therefore, miniaturization of LAMP is ideal in comparison with PCR. LAMP can be considered a promising technique for next-generation POC devices due to its excellent sensitivity (10–100 times higher than conventional PCR and 500–1000 times higher than rapid antigen and antibody detection), rapidity, resilience, and specificity; and its ease of use in real-world applications [181]. In this review, the recent developments on LAMP-based POC platforms integrated with microfluidic devices for rapid detection of infectious diseases and their detection mechanisms were explained in detail with suitable examples. Among the various detection methods, colorimetric and electrochemical detection methods are extensively used for LAMP amplicon detection. LAMP-based lateral flow immunoassay test kits also have emerged as rapid POC tests for various infectious pathogens, including COVID-19. Numerous smartphone-based fully automated devices have also been developed for sample-to-answer solutions. PDMS/PMMA, paper-based, and hybrid microfluidic chips are proven to be attractive tools for the development of the low-cost, rapid LAMP-based tests at POC. In addition, digital LAMP provides an accurate quantification of nucleic acid within the sample and eliminates the cross-contamination between samples. Furthermore, novel detection approaches such as bioluminescence [182] and giant magnetoresistance (GMR) [183] have been developed for the detection of LAMP reactions.

### 7.1. Alternative Methods

Enzyme-free isothermal amplification methods, such as hybridization chain reaction (HCR)-based POC sensors, could be an alternative technique. HCR works at room temperature; therefore, the use of a heating device is eliminated, making the overall operation process simple [184]. The amplified product can be detected by colorimetric, fluorometric, or electrochemical methods [185]. In recent years, the HCR method has emerged as a sensitive tool for infectious disease diagnosis [186,187,188]. Apart from that, other isothermal amplifications, including RCA and RPA, have been employed in multiplex detection with great sensitivity and versatile assay design [189,190]. RPA is performed with a temperature range of 32–42 °C and a rapid reaction time of 20 min [191]. RPA was also combined with LAMP to develop a hybrid assay in a microfluidic device for the simultaneous detection of 16 infectious pathogen nucleic acids, including ZIKA, HIV, and HPV [192]. Integration of these isothermal amplification methods in a microfluidic device can deliver a simple and rapid POC diagnostic tool for pathogenic nucleic acid detection.

### 7.2. Future Directions

LAMP is useful for diagnosing a wide range of pathogenic infections, including those of bacteria, viruses, and parasites. According to studies, the technique can detect and analyse pathogens in a sensitive, time-efficient, and POC manner. Nevertheless, there is still a lot of space for developments in LAMP-based POC diagnosis. Since LAMP is prone to producing significant nonspecific amplification, it is vital to develop novel methods for enhancing its specificity, particularly by utilizing nanomaterials that reduce nonspecific amplification [193]. To enhance sensitivity and performance, LAMP can be combined with other advanced diagnostic methods. For example, Pang et al. [194] developed a single-tube assay combining RT-LAMP with CRISPR/Cas12a for the diagnosis of COVID-19 targeting the N and E genes of viral RNA. The single-tube analysis was achieved by conducting RT-LAMP in the tube and loading the CRISPR Cas12a reagents in the lid of the reaction tube. After a 30 min RT-LAMP reaction, the CRISPR reagents were blended with LAMP amplicons by flipping the tube. The crRNA recognizes the specific sequence of the amplicons that initiated the Cas12a activity. A smartphone-based fluorescence analysis enabled the detection of SARS-CoV-2 with a LOD of 30 copies/µL in just 40 min. Furthermore, exothermic chemical-reaction-based electricity-free cartridges can eliminate reliance on electrical power and offer an accurate test without access to specialized facilities [189].

The entire workflow, from sample preparation to target identification, should ideally be compacted and integrated with the on-chip LAMP system. However, developing a fully automated sample-to-result microfluidic device requires the incorporation of micro-pumps and valves, which makes the chip fabrication and the operational process complicated. Microfluidic platforms made up of paper and capillaries make appealing substrates for LAMP amplification when creating straightforward, affordable molecular experiments, especially for applications in remote or resource-constrained locations. The paper/polymer-based hybrid systems are also capable of holding reagents in dried form for a long period, solving the problems of storing and transporting the reagents [195]. Microfluidic chip design with multiple reaction wells for an individual target can enable multiplex detection in a single reaction [130]. Droplet-based dLAMP can overcome carry-over contamination by sealing the individual sample droplets in oil [154]. Otherwise, one could design sealed microwells for amplification to circumvent the regent escape [159]. Droplet dLAMP can also allow multiplexing by separating the individual targets into separate droplets [158]. Overall, on-chip nucleic acid extraction, purification, and rapid sensing technology integrated with LAMP-based microfluidic modalities is the ideal candidate for POC diagnostics assays, capable of delivering high throughput and rapid pathogen detection in a multiplex manner, which reduces operation costs, analysis time, and carryover contaminations.

## Figures and Tables

**Table 1:** Comparisons of end-point detection methods of LAMP amplicons.

Technique	Pathogen	Signal Transduction Material	Readout Method	Time	LOD	Reference
Colorimetric	SARS-CoV-2	SYBR Green I	Visual	10 min	10^−3^ copies/reaction	[59]
	SARS-CoV-2	Phenol red		60 min	100 copies/reaction	[53]
	Rotavirus A	Neutral red		30 min	1 × 10^3^ copies/mL	[52]
	Six bacteria	HNB		60 min	10^2^–10^3^ CFU/mL	[48]
	HIV, HBV, HCV	Calcein		65 min	2 copies/µL	[47]
Lateral flow	*L. monocytogenes*	Fe_3_O_4_NPs	Visual	60 min	10 CFU/mL	[87]
	*S. typhimurium*	QBs-labeled LFIAS		60 min	10^3^ CFU/mL	[88]
	SARS-CoV-2	hCG-probe		120 min	0.5 copy/µL	[92]
Electrochemical	*V. parahaemolyticus*	Hoechst-33258	Differential pulse voltammetry (DPV)	45 min	0.3 CFU per 25 g raw seafood	[76]
	HPV	Benzoquinone (BQ)	Chronoamperometry	40 min	0.1 ng	[79]
	Group B *Streptococci*	Methylene blue	Cyclic voltammetry (CV)	45 min	0.23 fg/μL	[77]
	SARS-CoV-2	Methylene blue	Square wave voltammetry (SWV)	30 min	~2.5 × 10^−6^ ng/µL	[81]
Optical	*L. monocytogenes*	SYTO-9	CMOS	60 min	6 copies/μL	[97]
	*Salmonella typhi*	AuNPs	SPR	2 h	20 CFU/mL	[99]
	MRSA	Biotinylated-ssDNA probes	SPR	60 min	10 copies/μL	[98]
	*S. enterica*	AuNP-Cy5/DNA	SERS	40 min	66 CFU/mL	[100]
	*L. monocytogenes*	Multifunctional AuNPs	SERS	60 min	3.6 × 10^2^ CFU/mL	[101]
Diffusometric	*Vibrio cholerae*	400 nm green fluorescent particles	CMOS	35 min	6 cells/reaction	[106]
	SARS-CoV-2	400 nm green fluorescent particles	CMOS	35 min	35 × 10^4^ viral particles/mL	[108]
	*E. Coli*	1 µm Janus particles	CCD	10 min	42.8 fg/µL	[104]
	SARS-CoV-2-*nsp2* cDNA	1 µm Janus particles	CCD	10 min	70 ag/µL	[105]

## Data Availability

Not applicable.
